# Altered intestinal microbiome and metabolome correspond to the clinical outcome of sepsis

**DOI:** 10.1186/s13054-023-04412-x

**Published:** 2023-03-28

**Authors:** Silei Sun, Daosheng Wang, Danfeng Dong, Lili Xu, Mengqi Xie, Yihui Wang, Tongtian Ni, Weisong Jiang, Xiaojuan Zhu, Ning Ning, Qian Sun, Shuyuan Zhao, Mengjiao Li, Peili Chen, Meiling Yu, Jian Li, Erzhen Chen, Bing Zhao, Yibing Peng, Enqiang Mao

**Affiliations:** 1 Department of Emergency, Ruijin Hospital, Shanghai Jiao Tong University School of Medicine, No. 197 Ruijin ER Road, Shanghai, 200025 China; 2 Department of Laboratory Medicine, Ruijin Hospital, Shanghai Jiao Tong University School of Medicine, No. 197 Ruijin ER Road, Shanghai, 200025 China; 3 Clinical Research Center, Ruijin Hospital, Shanghai Jiao Tong University School of Medicine, Shanghai, 200025 China; 4 Faculty of Medical Laboratory Science, College of Health Science and Technology, Shanghai Jiao Tong University School of Medicine, No. 197 Ruijin ER Road, Shanghai, 200025 China

**Keywords:** Sepsis, Gut microbiota, Gut metabolites, *Enterococcus*, *Bacteroides*, Tryptophan metabolism

## Abstract

**Background:**

The gut microbiome plays a pivotal role in the progression of sepsis. However, the specific mechanism of gut microbiota and its metabolites involved in the process of sepsis remains elusive, which limits its translational application.

**Method:**

In this study, we used a combination of the microbiome and untargeted metabolomics to analyze stool samples from patients with sepsis enrolled at admission, then microbiota, metabolites, and potential signaling pathways that might play important roles in disease outcome were screened out. Finally, the above results were validated by the microbiome and transcriptomics analysis in an animal model of sepsis.

**Results:**

Patients with sepsis showed destruction of symbiotic flora and elevated abundance of *Enterococcus*, which were validated in animal experiments. Additionally, patients with a high burden of *Bacteroides,* especially *B. vulgatus*, had higher Acute Physiology and Chronic Health Evaluation II scores and longer stays in the intensive care unit. The intestinal transcriptome in CLP rats illustrated that *Enterococcus* and *Bacteroides* had divergent profiles of correlation with differentially expressed genes, indicating distinctly different roles for these bacteria in sepsis. Furthermore, patients with sepsis exhibited disturbances in gut amino acid metabolism compared with healthy controls; namely, tryptophan metabolism was tightly related to an altered microbiota and the severity of sepsis.

**Conclusion:**

Alterations in microbial and metabolic features in the gut corresponded with the progression of sepsis. Our findings may help to predict the clinical outcome of patients in the early stage of sepsis and provide a translational basis for exploring new therapies.

**Supplementary Information:**

The online version contains supplementary material available at 10.1186/s13054-023-04412-x.

## Background

Sepsis, which occurs when a severe infection leads to a violent inflammatory response in the host, is a life-threatening disease worldwide. In some cases, sepsis progresses to multiple-organ dysfunction syndrome, which worsens outcomes. Accumulating evidence has established strong interactions between gut microbiota dysbiosis and the pathogenesis of multiple diseases, including sepsis [1–3]. During critical illness, gastrointestinal transit changes, as well as unique medication and nutrition patterns, can drive dramatic changes in the gut microbiota [1]. Conversely, a disturbed microbiome provides a niche for the growth of pathogenic microbes, whose components can translocate across an impaired gut barrier and worsen the condition [4]. Although previous studies have focused on the microbial features of patients with sepsis, the role of bacteria found to be altered in sepsis remains unclear. Metabolic signatures of sepsis have been determined in serum, but metabolic changes in the gut during sepsis are poorly understood [5]. The microbiota is known to generate metabolites that affect physiological processes of the host [6, 7]. Therefore, to elucidate the composition of the gut microbiota and metabolites in sepsis and their association with disease progression, we conducted an observational cohort study of sepsis patients admitted to our intensive care unit (ICU). Stool specimens collected at the time of admission were used to generate microbial and metabolic profiles. Additionally, a rat model of sepsis was established to mirror the intestinal gene expression characteristics and to validate the microbial features of the patients. We anticipate that this study will shed light on the underlying biological functions of sepsis-related bacteria, leading to earlier prediction and improved treatment of this hazardous disease.

## Method

### Study design and sample collection

**Fig. 1:**
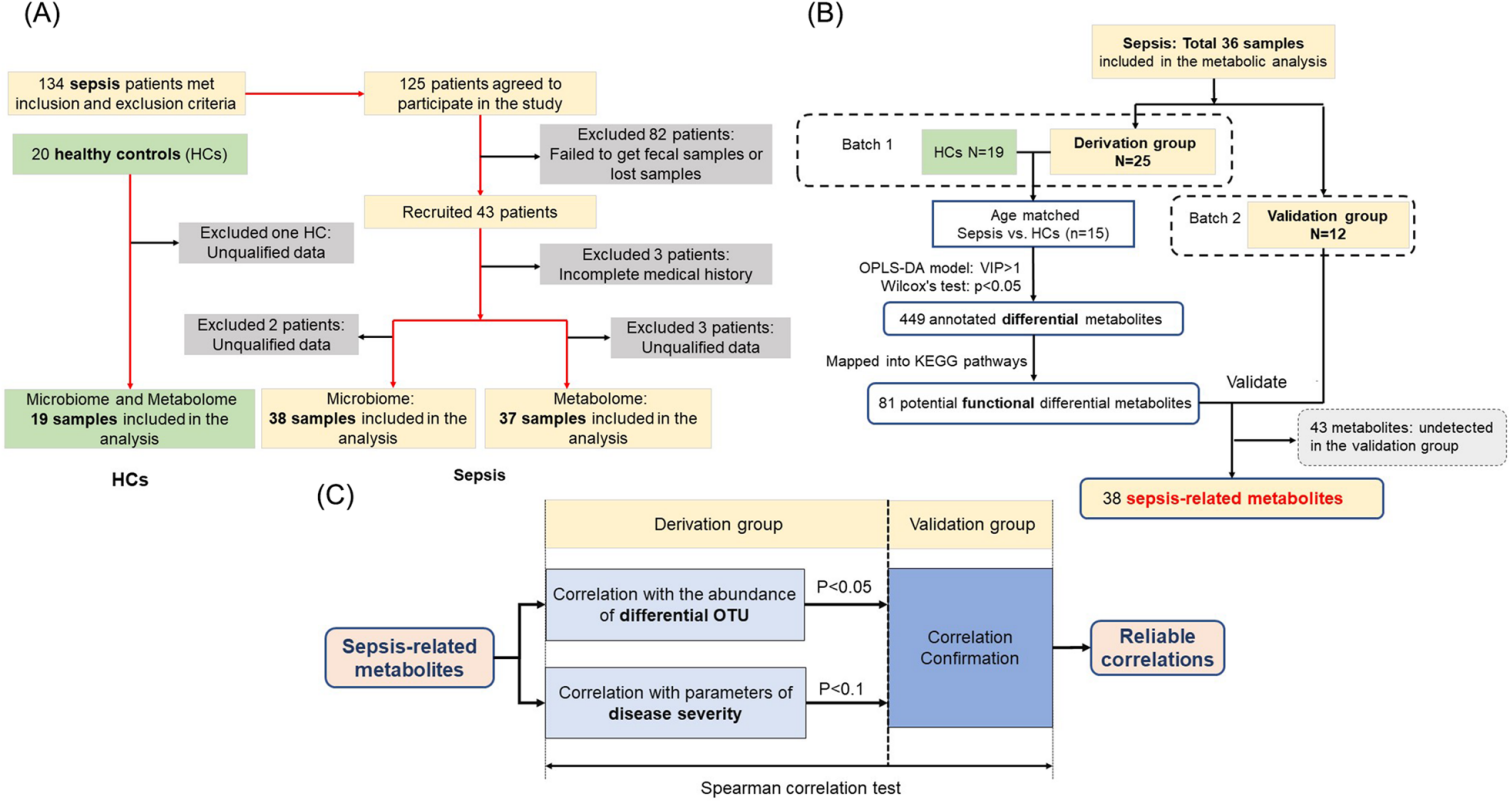
Flowchart outlining sample size calculation for the study. **A** Overview of the study design and patient group allocation. **B** Identification process for sepsis-related metabolites. **C** Identification process for reliable correlations in the metabolome analysis. HCs: health controls

This study is descriptive and observational, carried out with patients from a single center in China (Shanghai, China). Sepsis was defined in accordance with recommendations from the Third International Consensus Definitions for Sepsis and Septic Shock (Sepsis 3) [8]. Patients with terminal illnesses as well as those undergoing colostomy or ileostomy were excluded. As illustrated in Fig. 1A, 134 patients diagnosed with sepsis met the inclusion and exclusion criteria admitted to the Emergency Intensive Care Unit (EICU) of Ruijin Hospital from November 2019 to December 2021. Informed consent was obtained from patients or their relatives. Of them, 125 patients agreed to participate in this study. Sequential Organ Failure Assessment (SOFA) and Acute Physiology and Chronic Health Evaluation II (APACHE II) models were used to quantify the severity of patients on day 0 after admission. Basic information was also collected from each patient, including age, gender, antibiotic usage before admission, the origin of infection, proton-pump inhibitor usage, the time interval from medication to sample collection, complications during the ICU stay, mortality within 90 days, and number of days in the ICU. We collected stool specimens from a total of 43 patients, but after excluding 3 patients with incomplete medical histories, we ultimately enrolled forty patients. Additionally, 20 outpatients without any gastrointestinal disease or antibiotic treatment in the past month were randomly selected as healthy controls (HCs). The present study was approved by the Ethics Committee of Ruijin Hospital, Shanghai, China. More details on the cohort of patients can be found in Additional file 2.

After filtering for sequencing quality, we included thirty-eight sepsis patients and nineteen HCs in our microbiota comparison (Fig. 1A). Among them, thirty-seven sepsis samples met the quality requirements for metabolic analysis and were divided into two groups: the derivation group (*n* = 26) and the validation group (*n* = 12). The clinical characteristics of both groups were comparable, although the validation group had a higher proportion of male patients (Additional file 8: Table S1). We analyzed the gut metabolism of sepsis patients in the derivation group together with that of HCs in one batch, which allowed us to identify functionally annotated differential metabolites and their latent associations with microbes or clinical parameters. The metabolome of the validation group was analyzed separately, and only functional differential metabolites detected in this group were determined as sepsis-related metabolites. Moreover, only significant correlations confirmed in the validation group were defined as reliable correlations, based on the following criteria: (1) the direction of correlation was the same in both groups and (2) Spearman *r* < − 0.2 or > 0.2 (Fig. 1B, C).

### Specimen collection and processing

Immediately following defecation, stool samples were collected from patients, frozen at − 20 °C, and transferred to a − 80 °C freezer for long-term storage. Each specimen was divided into two parts (if applicable): one part for intestinal microbial composition analysis via 16S rRNA sequencing and the other for gut metabolomic profiling via untargeted liquid chromatography–mass spectrometry analysis. Microbiota and metabolite analyses were performed by Majorbio (Shanghai, China) and are described in further detail in the Additional file 1. The raw data were deposited into the NCBI Sequence Read Archive database (Accession Numbers: SRP407700, SRP407437).

### CLP model and RNA-sequencing

Male Sprague–Dawley rats at 8–12 weeks of age were obtained from the Shanghai Laboratory Animal Center of the Chinese Academy of Science (Shanghai, China). All rats were housed in laboratory conditions (25 °C, 12 h/12 h light/dark cycle, 50% humidity) and had free access to water and food. Animal procedures were conducted in compliance with the Animal Use and Care Committee of the Shanghai Committee on Animal Care. All surgical procedures were approved by the Institutional Animal Care and Use Committee at Shanghai Jiao Tong University. The CLP sepsis model was established in anesthetized rats, as previously described [9]. Briefly, rats were anesthetized with 1.5% pentobarbital (0.5 mL/100 g body weight) and a midline abdominal incision of 2.5 cm in length was made. The cecum was carefully exposed and ligated at 1.5 cm from the end, and two punctures were made with a 14-gauge needle. The abdominal wall was closed after returning the cecum to the abdominal cavity. Sham-operated rats underwent the same procedure, but without ligation and puncture. At 24 h after the induction of sepsis, all rats were killed. Colon segments containing feces were collected, immediately stored at − 80 °C, and subsequently used for 16S RNA sequencing to detect intestinal flora. Additionally, the distal ileum, and colon tissues taken from a fixed position of the rats, was collected, gently washed with normal saline, and stored at − 80 °C for subsequent transcriptome detection. Total RNA was isolated from small and large intestinal tissues of rats using the MiniBEST Universal RNA Extraction Kit (TaKaRa, Kusatsu, Shiga, Japan), and RNA sequencing was performed by Shanghai Personalbio (Shanghai, China). Transcriptomic analysis was performed by Majorbio (Shanghai, China). More details are provided in Additional file 1. The raw data were deposited into the NCBI Sequence Read Archive database (Accession Number: SRP407158).

### Statistical analysis

#### Clinical indicators

The results are expressed as means and standard deviations for continuous variables, and as frequencies and percentages for categorical variables. Student t-tests were used to compare differences in age and length of ICU stay. The proportions of APACHE II scores ≥ 18, SOFA scores ≥ 10 and ICU stay days ≥ 30, as well as mortality rates among enterotypes, were compared via Fisher’s exact test. These analyses were performed with GraphPad Prism 9.

#### Gut microbiota

Comparisons of the alpha diversity (Shannon and Chao indexes) of the gut microbiota were conducted using Student’s t-tests at the operational taxonomic unit (OTU) level. Principal coordinates analysis (PCoA) of the Bray–Curtis distance metric was performed to reflect the microbial structure of each sample, and dissimilarities were revealed through analysis of similarities (ANOSIM) testing. To assess the potential effects of covariates on the microbiota of sepsis patients, we compared the groups for Shannon index, principal component (PC)1 and PC2, *Enterococcus* OTU808 load, and *Bacteroides* OTU773 load using Student’s t-test or the Wilcoxon test through IBM SPSS Statistics 24.0. Linear discriminant analysis effect size (LEfSe) was evaluated from the taxonomic family level to OTU level, and different thresholds for linear discriminant analysis (LDA) scoring were set on various conditions. The predominant taxa were also compared among groups using the Wilcoxon test. Correlations between the abundance of an OTU and a clinical parameter or gene expression level were analyzed using Spearman’s correlation analysis, where a *p* value < 0.05 was considered significant. The correlation between the relative abundance of OTU773 and ICU stay days was also analyzed by multiple linear regression in two models.

#### Gut metabolites

The Wilcoxon test and fold changes analysis were performed. Metabolites with significant differences were selected based on the variable importance in projection (VIP), which was obtained from the orthogonal least partial squares discriminant analysis model, and the *p* value of the Wilcoxon test (threshold of VIP > 1, *p* < 0.05). Principal component analysis (PCA) was used to evaluate the interactive validation of specimens. The biochemical pathways of differential metabolites were identified by searching the Kyoto Encyclopedia of Genes and Genomes (KEGG; http://www.genome.jp/kegg/) database and classified by their pathway involvement. Enrichment analysis was conducted according to the presence of metabolites in a functional node. The scipy.stats Python package (https://docs.scipy.org/doc/scipy/) was used to test the statistical significance of enriched pathways identified by Fisher’s exact test. Spearman’s correlation analysis was employed to clarify associations between the abundance of metabolites, bacterial load, and indicators of clinical severity. Additionally, in order to assess the potential effects of covariates on the metabolome of sepsis patients, we compared the groups for principal component (PC)1 in both negative and positive mode using a Student’s t-test with IBM SPSS Statistics 24.0.

#### Gut transcriptome

Differential gene expression analysis was performed using both the edgeR and DESeq2 R packages, and Kal’s test with false discovery rate correction was applied. After Benjamini–Hochberg post hoc correction, an adjusted *p* value of < 0.05 was used for identification of differentially expressed genes (DEGs). Functional groups of DEGs were analyzed for involvement in KEGG pathways. A PCA plot was generated and used to assess variations in their interactions. To determine the core DEGs, a gene interaction network including all of the DEGs was created by Search Tool for the Retrieval of Interacting Genes/Proteins (STRING) and then imported into Cytoscape Software 3.7.0 for visualization. We ranked the DEGs by their betweenness centrality, obtained via the plugin CytoNCA; only the top-ranked DEGs were then selected for further correlation analysis.

All enrichment and correlation analyses were performed using the free online Majorbio I-Sanger Cloud Platform (www.i-sanger.com).

## Results

Table 1 lists the demographic data and clinical characteristics of the sepsis cohort of patients. Intra-abdominal (50%) and pulmonary (26.3%) infections were the predominant origins of sepsis in our patients. The occurrence of complications, mortality within 90 days, SOFA score, APACHE II score, and ICU stay time were recorded as indicators of the severity of sepsis. Twenty-one (55.2%) samples were collected within 48 h of medical treatment. The majority (35/38, 92.1%) of patients had received short-term antibiotic treatment in a lower-grade clinic before being admitted to our ICU. Among the antibiotics, carbapenem was the most frequently used (20/38, 52.6%), followed by 3rd/4th generation cephalosporins (10/38, 26.3%).

**Table 1:** Clinical and demographic characteristics of study subjects

	Sepsis (*n* = 38)	HC (*n* = 19)
Age, mean ± SD, years	61.33 ± 16.12	45.31 ± 12.37*
Gender, male/female	22/16	10/9
Origin of infection		
Intra-abdominal	19 (50.0%)	
Pulmonary	10 (26.3%)	
Complication occurred over the ICU period	12 (26.3%)	–
SOFA, mean ± SD	6.23 ± 3.40	–
APAHCE-II, mean ± SD	14.43 ± 19.07	–
Samples collected within 48 h of treatment	21 (55.2%)	
Mortality within 90 days, frequency (percentage)	5 (13.1%)	–
ICU stay, days	16.83 ± 13.15	–

APACHE; acute physiology and chronic health evaluation; SOFA; sequential organ failure assessment

*The HC group has a significant younger age than the sepsis group. (*p* < 0.001, student’s t-test)

### Sepsis-related bacteria distinguished by microbiota comparison between sepsis patients and HCs

**Fig. 2:**
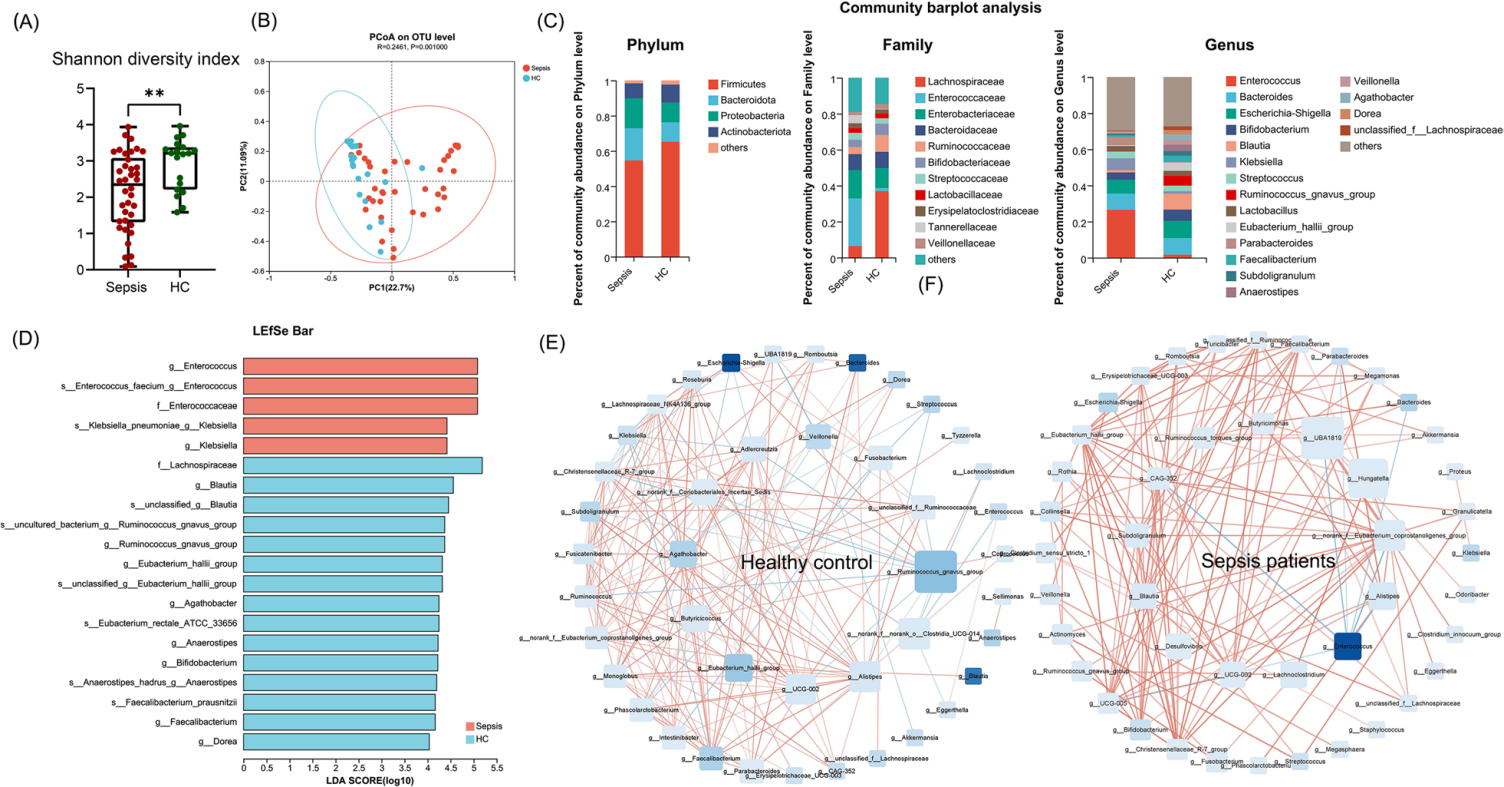
Comparison of microbial alterations in sepsis patients and HCs. **A** Student’s t-test showing differences in the Shannon diversity index of fecal samples from sepsis patients (*n* = 38) and HCs (*n* = 19). Data represent the median and quartiles  of each group; **p < 0.01. **B** PCoA of the sepsis and HC groups, with plots based on the Bray–Curtis distance. Each point represents a sample and the colors represent different groups. The results of the ANOSIM test to compare dissimilarity indexes among samples are shown above the plots. **C** Average relative proportions of the main phyla, families, and genera in the two groups. **D** LEfSe used to identify essential differences in bacterial abundance (family to species level) between the sepsis and HC groups. Only taxa with a significant LDA threshold value > 4 are shown. **E** Spearman’s correlation analysis to evaluate the abundance between the most common bacteria at the genus level in the two groups. Only correlations with a *p* value < 0.05 are shown. Color shading of nodes represents species abundance. Color gradation of lines represents the *R* value, with negative correlations shown in blue and positive correlations shown in red

First, we compared the alpha diversity and beta diversity between the sepsis and HC groups. Sepsis patients had obviously lower alpha diversity in the gut than HCs (Shannon index 2.146 vs. 2.911, *p* < 0.01; Fig. 2A) and a disparate microbial structure compared with the HC group (ANOSIM test, *p* = 0.001; Fig. 2B). The distribution of prevalent bacteria in sepsis and HC group is shown in Fig. 2C. Sepsis patients showed expansion of Proteobacteria phylum and *Enterococcaceae* family, but a remarkable drop in the proportions of Firmicutes phylum and *Lachnospiraceae* family. LefSe revealed that the sepsis patients also exhibited significant reductions in the abundance of health-promoting flora, such as *Blautia*, *Anaerostipes*, *Bifidobacterium*, and *Eubacterium_hallii_group* [10–13]. Conversely, *Enterococcus* and *Klebsiella*, the common nosocomial infectious agents [14], were more enriched in sepsis patients than in HCs (Fig. 2C, D). Furthermore, compared to HCs, sepsis patients had a less intricate network of inter-bacterial relationships, in which *Enterococcus* showed exceptional inhibitory effects on other taxa (Fig. 2E). Taking into account the age discrepancy between the two groups, we repeated these comparisons in an age-matched manner. After controlling for the effect of age, the Shannon diversity index was found to be similar between the sepsis group and the control group (Additional file 3: Figure S1A). However, the sepsis group still displayed a distinct microbial composition compared to the control group (Additional file 3: Figure S1B, ANOSIM test, *p* = 0.001), as evidenced by changes in the relative abundances of *Blautia*, *Eubacterium_hallii_group*, and *Enterococcus*, which remained statistically significant (Additional file 3: Figure S1C).

Concerning the differences in backgrounds and medications among the sepsis cohort, we also analyzed the potential influence of these variables on microbial structure (Additional file 9: Table S2). According to the results, age, gender, the time interval from medical treatment to specimen collection and the origin of infection had no significant effect on the microbiota of the sepsis group. Antibiotic type, number of antibiotics, and use of proton-pump inhibitors did not cause apparent changes in the microbiota in our sepsis cohort, which might be explained by the short exposure time to these medications. The body mass index (BMI), which can be related to distinct dietary habits, might be expected to have an effect on the microbial composition.

### Correlation between metabolic profile and the altered microbiota in patients with sepsis

**Fig. 3:**
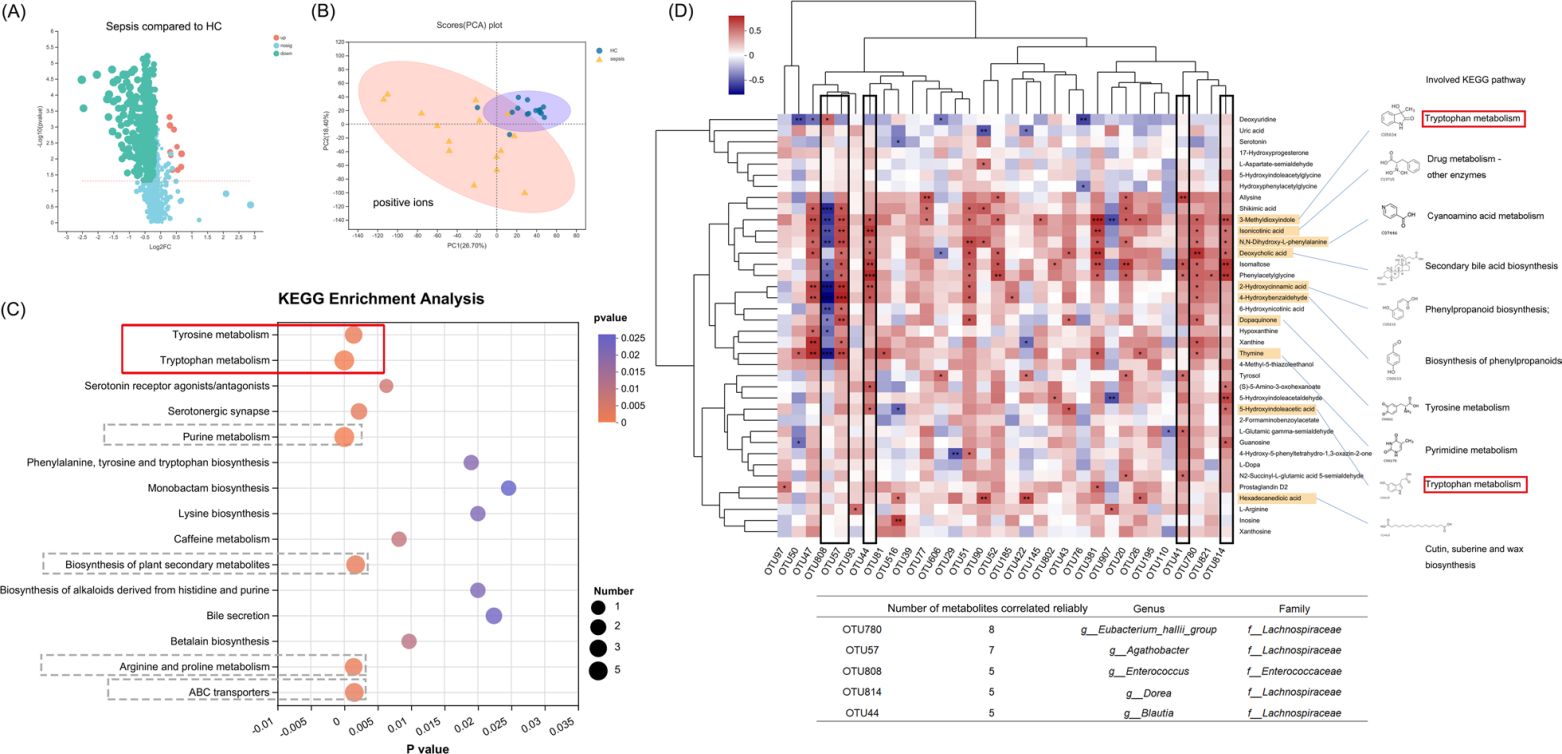
Differential gut metabolites of the sepsis patient cohort and their correlations with the microbiota. **A** Volcano plot showing the differential metabolites between sepsis patients and HCs. Green and red dots represent depleted and enriched metabolites, respectively, with the red line denoting a cut-off *p* value of < 0.05. **B** PCA of the stool samples from sepsis patients and HCs, plotted in positive-ion mode. **C** Bubble diagram illustrating the KEGG enrichment analysis. The size of each bubble represents the number of metabolites enriched in the pathway, and the color gradient indicates the significance of enrichment. Comparisons between the sepsis patients and HCs were observed after age matching (*n* = 15 per group). **D** Spearman’s correlation heatmap showing the test results for differential species in OTU level (LDA > 3.0) and sepsis-related metabolites among fecal samples in the derivation group (*n* = 25). Different colors indicate correlation level; *p < 0.05; **p < 0.01, ***p < 0.001. The 10 metabolites highlighted on the right were the most reliably correlated with microbial taxa after confirmation in the validation group

Next, we performed non-targeted metabolomic profiling of feces samples from sepsis patients and HCs. Although a total of 449 annotated differential metabolites were identified in the derivation group, only 10 (2.23%) were more abundant in the sepsis group than the HC group, suggesting a possible weakening of metabolic activity in these patients (Figs. 1B and 3A). PCA analysis revealed a distinct metabolic profile in both positive and negative ion patterns between the two groups (Fig. 3B, Additional file 4: Figure S2A). We assessed the impact of various variables on the metabolome and found that the number of antibiotics used may affect the metabolic composition (Additional file 9: Table S2, Additional file 4: Figure S2B). Furthermore, we mapped the sepsis-altered metabolites to their biochemical pathways using metabolic enrichment and pathway analysis based on KEGG annotations (Fig. 3C). Among the 81 potential functionally differential metabolites, 38 of them were detected in the validation group, which we defined as sepsis-related metabolites (Fig. 1B). We also evaluated the relationship between sepsis-related metabolites and other parameters among sepsis patients and considered only the correlations confirmed in the validation group to be reliable (Fig. 1C).

The differential metabolites appeared to be heavily involved in amino acid metabolism, including tryptophan, tyrosine, and arginine and proline metabolism. Involvement in imbalanced purine metabolism, biosynthesis of plant secondary metabolites, and ABC transporters was also observed. Considering the intimate contact between gut microbes and metabolites, we appraised the reliability of correlations between altered taxa and sepsis-related metabolites (Figs. 3D and Additional file 4: S2C). A variety of bacteria were found to be strongly associated with these key metabolites. Among the top five, four belong to *Lachnospiraceae*, indicating the potent metabolic regulatory function of this family of bacteria. Notably, OTU808 (*Enterococcus faecium*) showed an almost negative correlation with metabolites, which was similar to the above-mentioned inhibitory relationship between *Enterococcu*s and other bacteria in the intestinal microenvironment of sepsis. Furthermore, we identified the 10 metabolites most closely related to bacteria: 3-methyldioxyindole, 5-hydroxyindoleacetic acid, dopaquinone, *N*,*N*-dihydroxy-l-phenylalanine, isonicotinic acid, deoxycholic acid, 2-hydroxycinnamic acid, 4-hydroxybenzaldehyde, thymine, and hexadecanedioic acid. Six of these are aromatic compounds, which might be derived from metabolites of aromatic amino acid (AAA), including tryptophan, tyrosine, and phenylalanine. Among them, 3-methyldioxindole, a metabolite involved in tryptophan metabolism, showed the most relevance to gut microorganisms found in patients with sepsis.

### Agreement between gut microbial and metabolic structure and clinical severity of sepsis

**Fig. 4:**
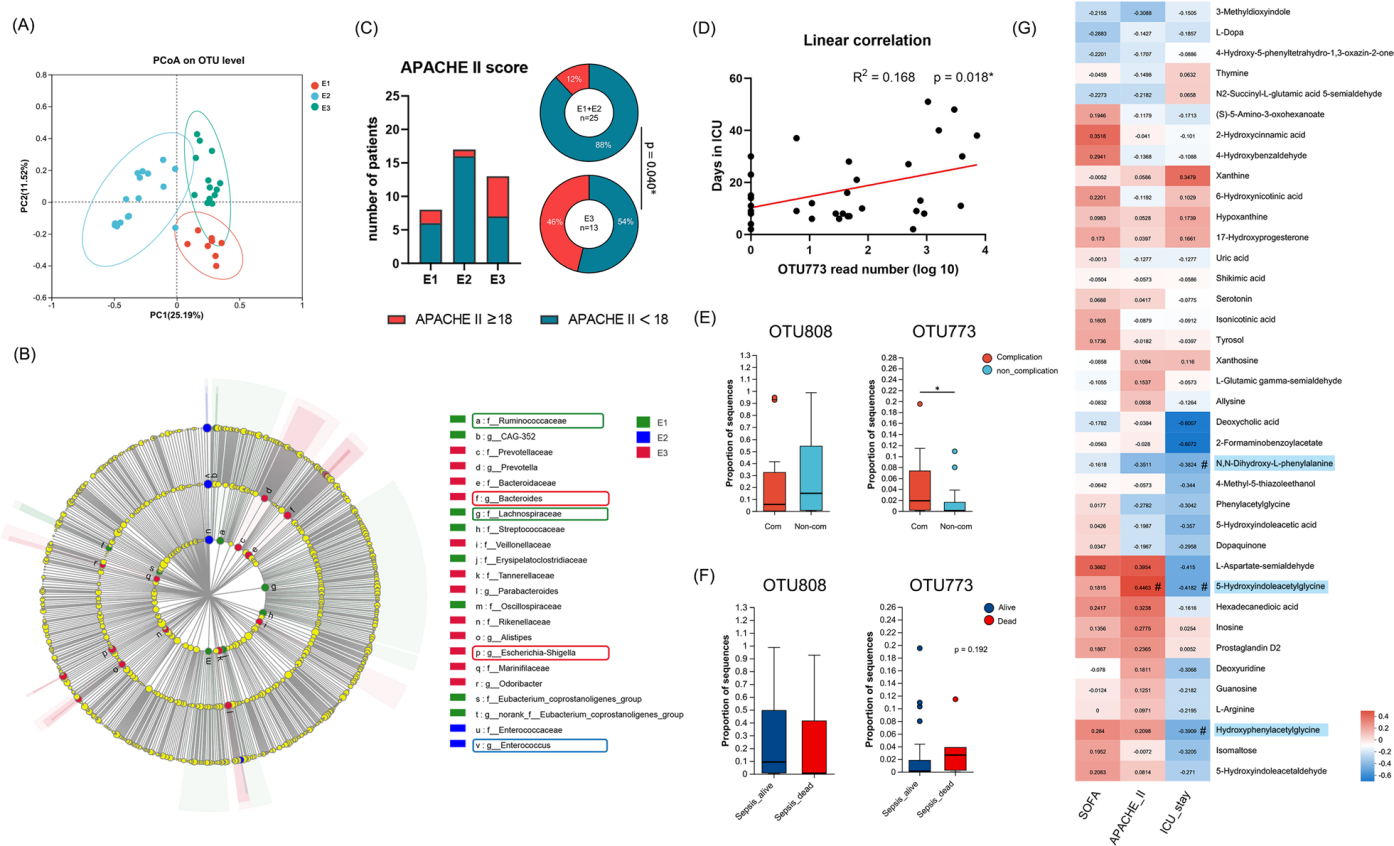
Parallels between microbial and metabolic profiles and clinical severity of sepsis. **A** PCoA of all sepsis patient samples (*n* = 38), with plots based on the Bray–Curtis distance. Each point represents a sample. According to the dispersion, the samples were classified into three enterotypes, shown in different colors. **B** LEfSe used to display the dominant bacteria in the three enterotype subgroups (family to species level): E1, *n* = 8; E2, *n* = 17; E3, *n* = 13. Only taxa with an LDA value > 4 are presented. Circles indicate phylogenetic levels; diameter and color of each circle represent its abundance and enterotype, respectively. **C** Column diagram showing the proportion of sepsis patients with an APACHE II score ≥ 18. **D** Linear correlation showing the relationship between the relative abundance of OTU773 and the length of ICU stay among surviving sepsis patients (*n* = 33). **E**, **F** Wilcoxon rank sum tests comparing the relative abundance levels of OTU808 and OTU773 between the sepsis patients with (*n* = 12) and without (*n* = 26) complications during their ICU stay (**E**), and between surviving (*n* = 33) and nonsurviving (*n* = 5) sepsis patients (**F**). **G** Spearman’s correlation heatmaps representing relationships between clinical parameters and the relative abundance of sepsis-related metabolites among surviving sepsis patients in the derivation group (*n* = 21). Distinct colors represent correlation levels; “#” indicates a reliable correlation

The potential effects of the gut microbiome and metabolites on the severity of sepsis were examined next. As shown in Fig. 4A, patients in this group could be generally classified into three subgroups based on their gut microbial composition, also known as enterotypes. *Lachnospiraceae* and *Ruminococcaceae* prevail in enterotype 1 (E1); enterotype 2 (E2) is dominated by *Enterococcus*; and enterotype 3 (E3) is abundant in *Bacteroides* and *Escherichia-Shigella* (Figs. 4B and Additional file 5: Figure S3A). The comparison of enterotypes revealed that patients in E3 were the most critical, with a significantly larger proportion of APACHE II scores ≥ 18 compared to non-E3 patients (46% vs. 12%, *p* = 0.040; Fig. 4C). Additionally, E3 patients had a potentially larger proportion of SOFA scores ≥ 10 and ICU stays ≥ 30 days, as well as the highest mortality rate, but these differences did not reach statistical significance (Additional file 5: Figure S3B, Additional file 10: Table S3). Although E2 patients appeared to have lower bacterial diversity and richness relative to the other enterotypes (Additional file 5: Figure S3C), their disease was not more severe than the others.

We also analyzed the correlations between the predominant bacterial taxa and clinical indices of surviving sepsis patients. Our analysis revealed that two OTUs, OTU773 from *Bacteroides vulgatu*s and OTU822 from Rikenellaceae (*s__unclassified_g__Alistipes*), were significantly positively correlated with the length of ICU stay (Figs. 4D and Additional file 5: Figure S3D). The correlation between *B. vulgatus* and the length of ICU stay remained significant even after removing the potential effect of confounding variables in a multiple linear regression model (Additional file 11: Table S4). On the other hand, OTU808 (*E. faecium*) showed only a negligible correlation with this clinical parameter. Additionally, patients who experienced complications during the ICU period had significantly higher OTU773 (*B. vulgatus)* loads, but did not exhibit differences in the OTU808 (*E. faecium)* loads (Figs. 4E and Additional file 5: Figure S3E). Although not significant, there was a similar trend showing that OTU773 (*B. vulgatus)* was more abundant among the patients who died from sepsis (Fig. 4F). These results suggested a possible adverse effect of *B. vulgatus* on sepsis patients. Furthermore, we found that other bacteria, such as *Akkermansia* and OTU40 from Micrococcaceae (*s__unclassified_g__Rothia*), might have exerted beneficial effects on our sepsis patients. OTU40 was inversely correlated with APACHE II scores, while the abundance of *Akkermansia*, a genus known to be protective in many human diseases, was lower in the gut microbiota of non-surviving sepsis patients (Additional file 5: Figure S3D, F) [15].

Assessment of the reliability of correlations between gut metabolites and the disease severity of sepsis uncovered three compounds that were negatively correlated with length of ICU stay (Table 2 and Fig. 4G). Furthermore, we observed that two sepsis-related metabolites were reduced in the non-surviving patients (Table 2 and Additional file 4: Figure S2D). Four of the five metabolites were aromatic compounds and involved in amino acid metabolism, particularly metabolism of tryptophan and tyrosine. Interestingly, 5-hydroxyindoleacetylglycine was also positively correlated with the APACHE II score, revealing an intricate network of interactions between gut-derived metabolites and sepsis (Fig. 4G). We also observed that non-surviving patients had an enriched abundance of kynurenine (Additional file 4: Figure S2E), which is also a tryptophan metabolite but was not detected in the validation group. Interestingly, we observed that the abundance of these tryptophan metabolites remained comparable in patients receiving different numbers of antibiotics, suggesting that the perturbation of tryptophan metabolism may not be associated with the number of antibiotics administered (Additional file 4: Figure S2F). Our results overall suggested that tryptophan metabolites played a pivotal role in the progression of sepsis.

**Table 2:** Important metabolites associated with the gut microbiota or clinical severity of sepsis patients

Metabolites	FCs in sepsis	Involved KEGG pathway	Associated OTU^a^	Spearman *r*			FCs in dead patients^c^
				SOFA	APACHE_II	ICU stay time^b^	
*Metabolites correlated with clinical parameters*							
N,N-Dihydroxy-L-phenylalanine	0.784	Cyanoamino acid metabolism	OTU808; OTU57; OTU44; OTU51; OTU381; OTU780; OTU814	− 0.162	− 0.351	− 0.382^#^	0.956
Hydroxyphenylacetylglycine	0.697	Tyrosine metabolism	– –	0.264	0.210	− 0.391^**#**^	0.913
5-Hydroxyindoleacetylglycine	0.672	Tryptophan metabolism	– –	0.182	0.446^#^	− 0.418^#^	0.844
*Metabolites significantly changed in non-surviving sepsis patients*							
5-Hydroxyindoleacetaldehyde	0.778	Tryptophan metabolism	OTU814	0.208	− 0.081	− 0.271	0.653*
Tyrosol	0.775	Tyrosine metabolism	–	0.174	− 0.018	− 0.040	0.737*

^a^OTUs significantly correlated with the abundance of metabolites were determined as: (1) *p* value < 0.05 in the derivation group (*n* = 26) and (2) showing the same correlation direction in the validation group (*n* = 12) with a Spearman *r* > 0.2 or < − 0.2

^b^The Spearman correlation between the abundance of metabolites and clinical parameters was conducted in the surviving sepsis patients (*n* = 22)

^c^The fold change was obtained relative to alive patients

“*” means significantly changed with a threshold of *p* value < 0.05;

“#” means the correlation not only possesses a *p* value < 0.1 in the derivation group but also remains the same direction with a Spearman *r* > 0.2 or < − 0.2 in the validation group

### Changes in the gut microbiota and intestinal gene expression in septic rats

**Fig. 5:**
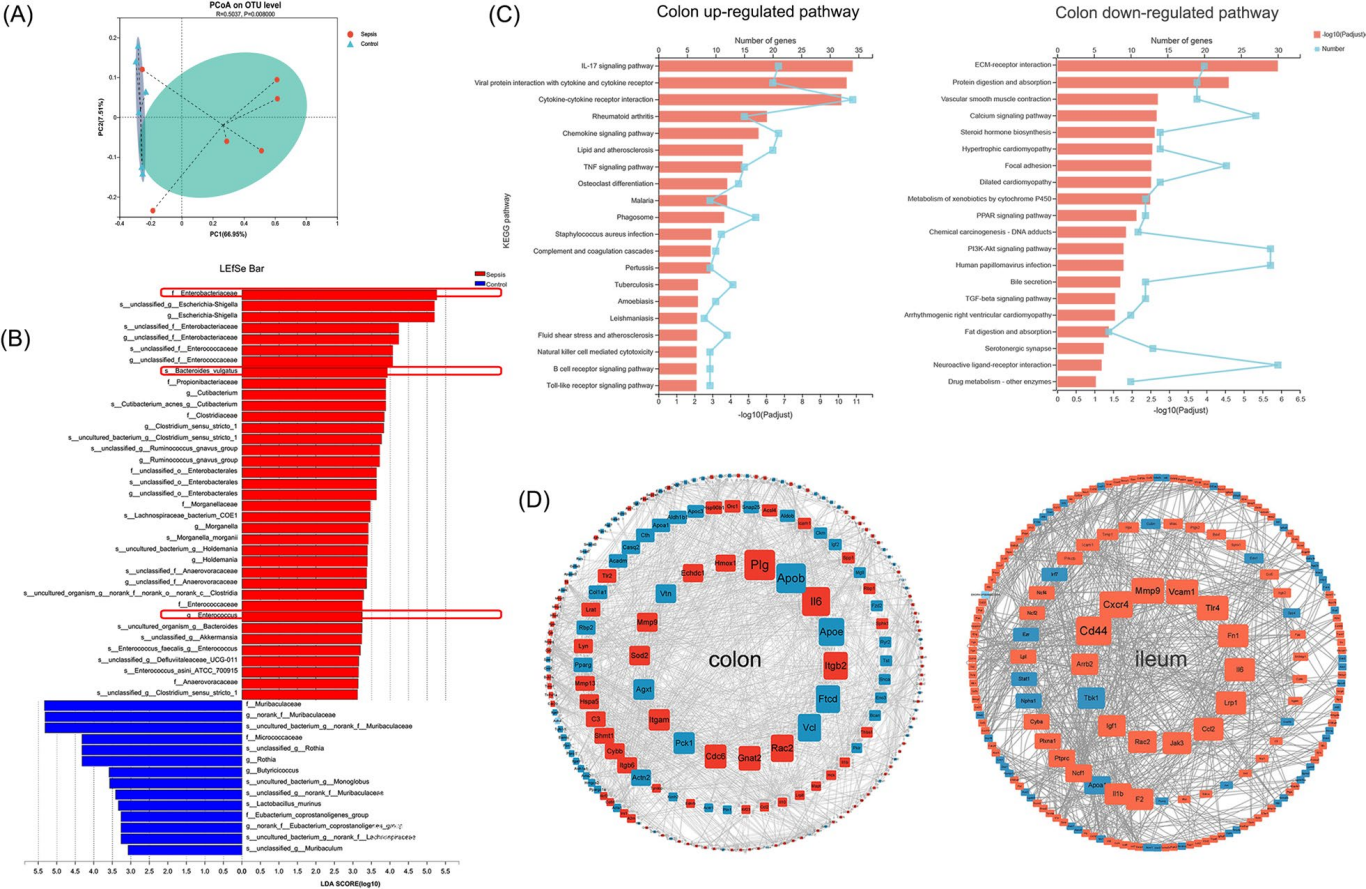
Bacterial portrait and intestinal transcriptomic adjustment in the septic rat. **A** PCoA of data from CLP sepsis model rats and control rats (*n* = 6 per group), with plots based on the Bray–Curtis distance. Each point represents one sample and the colors indicate different groups. The results of the ANOSIM test to compare dissimilarity indexes among samples are shown above the plots. **B** LEfSe performed to identify important differential bacterial taxa (family to species level) between the sepsis patient and HC groups (*n* = 6 per group). Only taxa with a significant LDA threshold value > 3 are shown. **C** KEGG pathway enrichment analysis of DEGs upregulated (left) and downregulated (right) in the septic colon (*n* = 6) relative to their expression in the colon of HCs. The top 15 pathways are shown. **D** Gene expression network generated by STRING and displayed via Cytoscape. Red (colon, left) and orange (ileum, right) rectangles represent upregulated genes and blue circles represent downregulated genes in the sepsis group. The size of each gene node is proportional to its BC value, and the higher the BC value, the closer the node to the center of the circle

Even if our assessment of stool specimens closely replicated the types of specimens that are routinely encountered in clinical practice, it is theoretically difficult to eliminate the interference of the multifarious clinical medications on the human intestinal microbiota. Therefore, we sought to corroborate the sepsis-related microbial features of our sepsis cohort of patients in an animal model. Having observed that our sepsis patients with intra-abdominal infection had a similar microbiome structure to those with lung infection (Additional file 3: Figure S1D), we established the classic CLP model of sepsis, which is caused by abdominal infection, in rats. Similar to the patient and HC groups, the septic rat group exhibited divergence from the sham operation group in the intestinal microbial composition (ANOSIM test, *p* = 0.008; Fig. 5A), although there was slight variation in the Shannon diversity index (Additional file 6: Figure S4A). Likewise, the proportions of essential probiotic bacteria, including Muribaculaceae and *Butyricicoccus*, dropped in septic rats, while opportunistic pathogens like Enterobacteriaceae and *Enterococcus* prospered (Fig. 5B) [16–18]. Notably, the abundance of *B. vulgatus,* which was associated with poor outcomes in our sepsis patients, was increased in the intestine of septic rats, despite the comparable loads of *B. vulgatus* in the stool specimens of sepsis patients and HCs.

To further illuminate the functions of key sepsis-related bacteria, we further evaluated the relationship between the bacterial abundance profile and the intestinal gene expression profile. Initially, we focused on transcriptomic variance in the septic intestine of rats. KEGG analysis was conducted to determine the functional enrichment of DEGs (Fig. 5C, Additional file 6: Figure S4B). For both the ileum and colon, masses of genes that were upregulated in sepsis were found to be involved in cytokine–cytokine receptor interaction and the chemokine signaling pathway, as reflected by the immune activation. Multiple cell adhesion molecules were downregulated in the septic colon, as illustrated by the weak functioning of the extracellular matrix (ECM)–receptor interaction with the transforming growth factor-β signaling pathway, which contributes equally to the recruitment of immune cells and the enhancement of intestinal permeability [19–21]. Another notable alteration was found in the lipid metabolism pathway. Numerous genes that were downregulated are pertinent to steroid hormone biosynthesis, the peroxisome proliferator-activated receptor (PPAR) signaling pathway and cholesterol metabolism. Cholesterols are precursors for several hormones that exert an anti-inflammatory function, and the PPAR signaling pathway can be activated by fatty acids and their derivatives, which effectively ameliorate inflammatory damage in sepsis [22–24]. Additionally, the pathway related to the metabolism of xenobiotics was also diminished in sepsis. This pathway responds to the activation of aryl hydrocarbon receptor (AhR) and pregnane X receptor (PXR) by gut-derived metabolites and subsequently interacts with nuclear factor-κB signal transduction to exert an anti-inflammatory function [25].

Next, a protein interaction expression network of the DEGs was conducted through STRING. The central DEGs are shown in Fig. 5D. Regarding core DEGs in the colon, *Plg* assists with ECM disruption and neutrophil migration, *Il6* is involved in inflammation, and *Apob* and *Apoe* are components of lipoprotein carriers, which might modulate the immune process by binding endotoxin or other toxins [26, 27]. Regarding DEGs in the ileum, *Cd44*, *Vcam1* and *Cxcr4* serve the immune cell aggregation process, while *Mmp9* degrades adhesive substances to promote cell movement [28–31]. Taken together, the intestinal transcriptome of sepsis revealed an inflammatory storm associated with various types of host response.

### Distinct interactions of specific bacteria in the intestinal transcriptome of septic rats

**Fig. 6:**
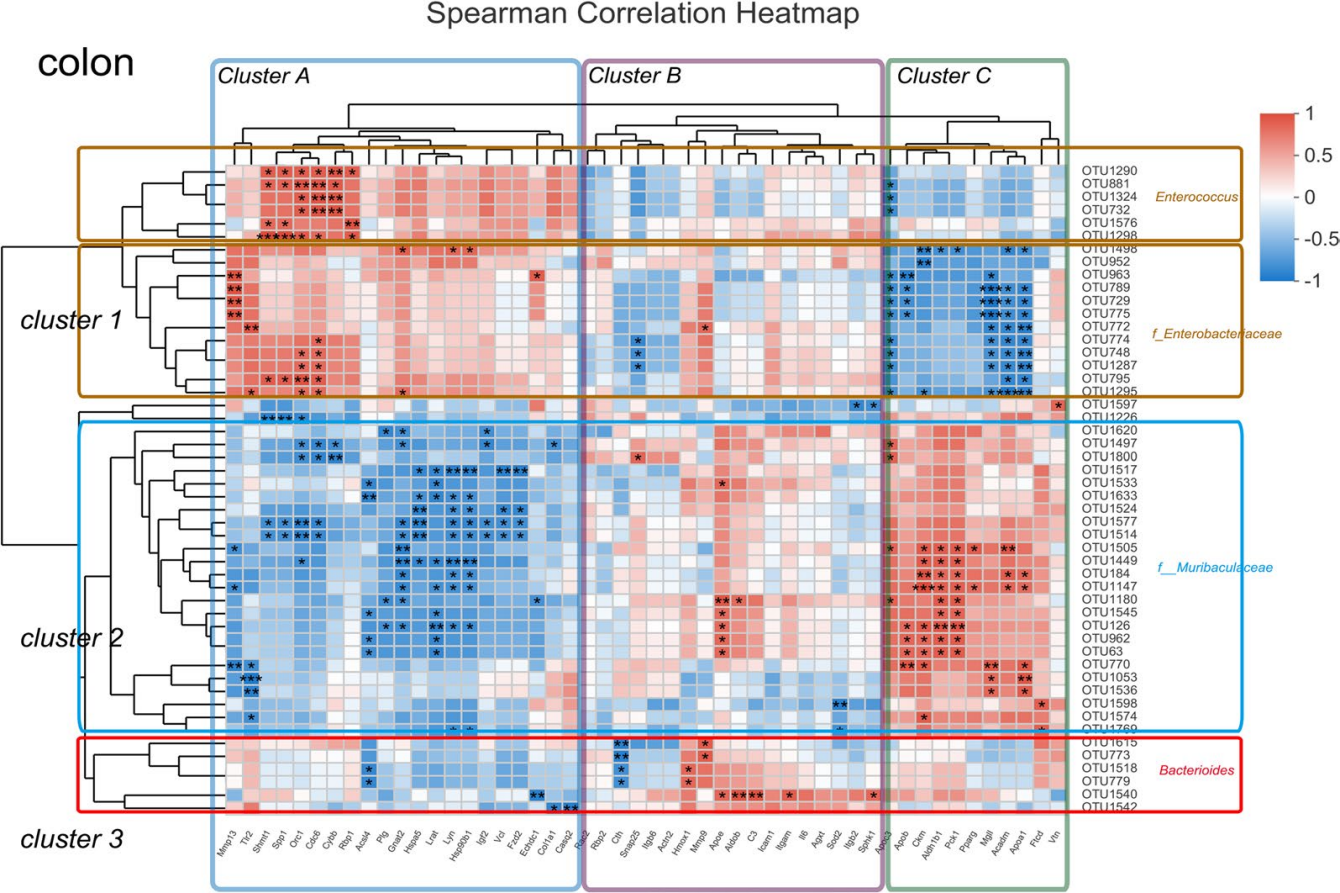
Correlation analysis of differential species and central DEGs in the colon of CLP model rats. Spearman’s correlation analysis conducted to evaluate associations between the top 50 central DEGs (identified by BC value) and differential OTUs (LDA > 2) in the colon of septic rats (*n* = 6). The color gradient corresponds to the *R* value, where red represents the highest positive correlation and blue represents the lowest

Earlier, we proposed that *Bacteroides* and *Enterococcus* are important sepsis-related bacteria that likely have different effects on disease progression. Therefore, we carried out a correlation analysis of microbial abundance and intestinal gene expression to identify the potential roles of gut microbes in the pathophysiology of sepsis. The results for the colon are shown in Fig. 6. We were able to distinguish three bacterial clusters (clusters 1–3) and three gene clusters (clusters A–C). Cluster 1 chiefly contained *Enterococcus* and Enterobacteriaceae and showed a positive correlation with genes of Cluster A, which function in inflammatory response (*Cybb*, *Tlr2*) and ECM disruption (*Mmp13*, *Spp1*). Furthermore, Cluster 1 organisms from Enterobacteriaceae are more closely related to genes relevant to lipid metabolism (*Apoc3*, *Apob*) than those from *Enterococcus*. Cluster 2, which comprised Muribaculaceae, showed an utterly different association trend compared with Cluster 1. *Bacteroides* species gathered in Cluster 3 and interacted with a few genes from Cluster B outstandingly. Among these gene interactions, *Mmp9,* a well-studied metalloprotease involved in barrier disruption, was positively correlated with *Bacteroides* species, while *Cth,* which encodes cystathionine-γ-lyase, a major endogenous hydrogen sulfide-producing enzyme shown to suppress inflammation through IL-8 signaling [22, 23], was negatively correlated. Correlation analysis of the ileum also revealed three main bacterial clusters and three gene clusters (Additional file 6: Figure S4C). The compositions of Clusters 1 and 2 were similar to their colonic counterparts and were tightly correlated with DEGs from Clusters A and B, but in the opposite direction. Although Clusters A and B were both composed of immune and ECM genes, they failed to show any distinct effects in sepsis. The majority of *Bacteroides* species were still found in Cluster 3. Only a few significant correlations were found for *Bacteroides*, of which the most remarkable was a link with *Cyba* expression. Ultimately, the correlation analysis revealed that *Bacterioides* and *Enterococcus* might influence different host intestinal physiological processes in sepsis.

## Discussion

Overall, we found that the microbial composition differed between a healthy condition and sepsis in both clinical patients and rats and was characterized by the loss of beneficial flora and overgrowth of potentially pathogenic bacteria. These results were consistent with previous findings in various cohorts of critically ill patients [1, 32]. In this study, we observed that *Enterococcus* was enriched in both patients and rats with sepsis and was accompanied by the flourishing of other potential pathogens, including *Klebsiella* in the patient cohort and Enterobacteriaceae in the CLP rats. Additionally, the abundance of *Enterococcus* and Enterobacteriaceae was associated with high colonic expression of several inflammatory genes in the animal model. A flourishing *Enterococcus* population often implies the fragile condition of the gut microbiota. Previous studies demonstrated that the elevated abundance of *Enterococcus* on admission was predictive of death or infection among ICU patients and was predictive of death from severe SARS-CoV-2 infection [33–35]. Moreover, our results demonstrate that *Enterococcus* exhibits a potent inhibitory association with most microbes and altered metabolites in sepsis patients. This phenomenon may be partially attributed to the competitive growth advantage of *Enterococcus* compared to metabolic activists. Previous studies have illustrated that *Enterococcus*, particularly vancomycin-resistant *Enterococcus* (VRE), can easily outcompete other bacteria due to their tolerance to starvation and antibiotic pressure [36, 37]. *Enterococcus* is also known to reconstitute the metabolic environment in the gut through the arginine deiminase pathway, which supports the growth of several pathogens, such as *Escherichia coli* and *Clostridioides difficile*, leading to a possible secondary infection [38, 39].

Although *Enterococcus* had multiple adverse effects on the gut environment in sepsis, we determined that *Bacteroides*, particularly *B. vulgatus,* was the key bacteria associated with the detrimental outcome of sepsis. Consistent with our results, research on sepsis patients in China led Liu et al. to propose that patients with an enterotype comprising predominantly *Bacteroides* rather than *Enterococcu*s were more likely to progress to septic shock [40]. Thus, it is conceivable that *Bacteroides* and *Enterococcus* played distinctly different roles in promoting sepsis in this study. Consistently, we observed that *Bacteroides* and *Enterococcus* were associated with divergent intestinal gene clusters in septic rats. Compared to *Bacteroides*, *Enterococcus* was positively correlated with more genes involved in cell chemotaxis and inflammatory damage and was negatively correlated with more genes involved in lipid metabolism. By contrast, *Bacteroides* might affect intestinal barrier permeability or H_2_S synthesis in the process of sepsis. Mills et al. demonstrated that *B. vulgatus* exhibited high protease activity and worsened barrier destruction in ulcerative colitis, consistent with its correlation with high expression of *Mm9* in sepsis [41]. This suggested that protease inhibitor treatments used for colitis might provide medicinal value for sepsis as well. Conversely, the function of *Bacteroides* in the host intestine remains controversial, with some research recognizing it as protective bacteria in several diseases, including obesity, atherosclerosis, and lipopolysaccharide-induced acute intestinal injury [42–44]. More experiments to clarify the precise mechanism of *Bacteroides spp.* in sepsis should be conducted in the future. We did not detect a close relationship between the *Enterococcus* load and clinical outcomes in this study, which could be explained in some aspects. Although Agudelo-Ochoa et al. discovered that sepsis patients with an increased *Enterococcus* burden during their ICU stay were less likely to survive, the dynamics of microbial composition are seldom considered in the evaluation of sepsis patients presently [34]. We propose that while *Enterococcus* bacteria may be destructive, their abundance in patients with sepsis at the start of ICU admission is probably less predictive of negative outcomes than that of *Bacteroides*. Moreover, the *Enterococcus* species that exhibited overgrowth in our patients was mostly *E. faecium.* In contrast with the notorious *E. faecalis*, *E. faecium* is sometimes seen as a probiotic because it has the capacity to secrete bacteriocins that limit bacterial pathogenesis. A plausible explanation for this result is that *E. faecium* thrives as well as *E. faecalis* in the septic environment, but leads to fewer adverse effects [45]. In any case, more precise discrimination will increase the reliability of prediction of disease course based on gut microbes.

The drop in intestinal bacterial diversity might have been partly responsible for the apparent scarcity of gut metabolites that we observed in sepsis patients. Consistent with previous findings based on serum specimens [5], metabolites involved in aromatic amino acid metabolism were the most interrupted. Our sepsis cohort exhibited reductions in a variety of aromatic amino acid metabolites, which are also closely linked with the gut microbiome and the outcome of sepsis, especially those involved in tryptophan and tyrosine metabolism. On the one hand, tryptophan and tyrosine are precursors of certain neuroactive compounds, including serotonin and noradrenaline, that modulate several cognitive disorders; deficiency of these amino acids is an important factor that prolongs the length of hospital stay among critically ill patients [46, 47]. On the other hand, tryptophan can also be converted to indole derivatives by several intestinal microorganisms, which are able to activate the AhR or PXR of intestinal epithelium cells or lymphocytes to benefit anti-inflammation, antimicrobial peptide secretion, and xenobiotic metabolism [48]. Among the sepsis-related metabolites identified here, some were previously shown to be AhR ligands (e.g., 5-hydroxyindoleacetic acid), and others were shown to be downstream metabolites of known AhR ligands (e.g., 3-methyldioxyindole) (Additional file 7: Figure S5) [49, 50]. Interestingly, *B. vulgatus* showed a weak correlation with tyrosine and tryptophan metabolites, suggesting that it may exacerbate sepsis independently (Additional file 4: Figure S2H). Additionally, tryptophan was metabolized by the host via the serotonin pathway as well as the kynurenine pathway [46]. In the present study, we observed that the metabolites of the serotonin pathway, which included 5-hydroxyindoleacetylglycine and 5-hydroxyindoleacetaldehyde, may have manifested beneficial effects, whereas kynurenines seemed to play aggravating roles. It is possible that proinflammatory cytokines activate indolamine-2,3-dioxygenase, the essential enzyme for conversion of tryptophan to kynurenine, which consumes all of the tryptophan that would normally be available for serotonin formation, thereby aggravating the cognitive impairment in sepsis [46]. This would also support the hypothesis that an imbalance of gut metabolites in the serotonin and kynurenine pathways is predictive of poor outcomes in sepsis.

We also identified several bacteria with potentially beneficial effects on the progression of sepsis. For instance, Lachnospiraceae species were found to be closely related to sepsis-related metabolites, including tryptophan metabolism. Some bacteria directly metabolize tryptophan, while others produce short-chain fatty acids (SCFAs) that modify the microbial structure and thereby indirectly facilitate tryptophan metabolism [48, 49, 51]. SCFAs are a group of metabolites that derive from bacterial fermentation of fiber and nourish gut epithelial cells to maintain intestinal integrity and potentiate intestinal anti-inflammatory processes [7]. Muribaculaceae bacteria have been reported to generate SCFAs, which might explain their inhibitory effects on intestinal inflammatory gene expression in rats [16]. The relationship between *Akkermansia* load and sepsis outcome also might be explained in part by its production of SCFAs [15]. Overall, precise optimization of the abundance of such bacteria might be effective for alleviating sepsis.

This study has several limitations. First, it has a small sample size, with all participants originating from a single health center; thus, our results should be verified in patients from other institutions. Second, although our findings remained significant when controlled for age, and were verified in the rat model, clinical confounders remained, including pre-ICU medications and the pathogens of infection. Third, the effects of *Enterococcus* and *B. vulgatus* in sepsis were inferred by correlation analysis and should be verified by experiments on animals colonized with specific bacteria. We suggest that future studies be conducted using metagenomics technology to precisely decipher the functions of microbial genetic elements involved in the alteration of gut metabolites in sepsis.

## Conclusion

In this study, we clarified the portrait of the gut microbiota in sepsis, correlating it with intestinal metabolites and the intestinal transcriptome to highlight potential effects on this devastating disease. Sepsis patients manifested deterioration of the intestinal microbiome characterized by depletion of important commensal bacteria and domination by *Enterococcus*. We have delineated, for the first time, the disruption of amino acid metabolism occurring in the intestines of sepsis patients. Specifically, we observed a sharp decrease in tryptophan and tyrosine metabolites that was closely associated with an altered composition of bacteria and more severe clinical outcomes. The microbial composition also impacted the severity of sepsis in patients, with *Bacteroides spp.* playing a predominant role in shaping the intestinal transcript profile, which differed from previous studies showing *Enterococcus* as the most common sepsis-related bacteria. It is essential that we gain a better understanding of the microbes and metabolites that contribute to the clinical outcomes of sepsis if we are to meet this urgent public health challenge.

## Supplementary Information

**Additional file 1:** Supplemental methods

**Additional file 2:** Clinical data of patients in the sepsis cohort

**Additional file 3: Figure S1.** Comparison of microbial alterations in the sepsis patients and the HCs after age matching (*n* = 17 per group). **A** PCoA for the sepsis and HC group samples, with plots based on the Bray–Curtis distance. Each point represents one sample and the colors represent different groups. The results of the ANOSIM test to compare dissimilarity indexes among samples are shown above the plots. **B** Wilcoxon rank sum tests performed to analyze between-group differences in the main bacterial load at the genus level. **C** Average relative proportions of genera in patients according to origin of infection.

**Additional file 4: Figure S2.** Metabolic profiles of sepsis patients and their association with gut microbiota and disease severity. **A** PCA of the stool samples from sepsis patients and HCs, plotted in negative-ion mode. **B** Principal component analysis (PCA) for septic samples receiving different numbers of antibiotics in positive-ion mode. **C** Spearman’s correlation heatmap showing the test results between differential species at the OTU level and altered sepsis-associated metabolites among samples in the validation group (*n* = 12). Distinct colors represent correlation level; *p < 0.05; **p < 0.01; ***p < 0.001. **D** Heatmap showing the significant differential metabolites between surviving (*n* = 21) and non-surviving (*n* = 4) patients in the derivation group. **E** Wilcoxon rank sum test performed to compare the abundance of fecal kynurenine between the surviving and nonsurviving patients. **F** Spearman’s correlation between the OTU773 (*B. vulgatus*) load and the abundance of sepsis-associated metabolites among samples in the derivation group (*n* = 25). Confirmation of the correlations between OTU773 and four metabolites (2-hydroxycinnamic acid, 4-hydroxybenzaldehyde, hypoxanthine, and L-glutamic gamma-semiarid) in the validation group (*n* = 12). Distinct colors represent correlation level; *p < 0.05; **p < 0.01; ***p < 0.001.

**Additional file 5: Figure S3.** Possible relationships between enterotype and clinical severity of sepsis. **A** Average relative proportions of genera in each enterotype. **B** Column diagrams showing the proportions of sepsis patients with a SOFA score ≥ 10, an ICU stay ≥ 30 days, and death. **C** Student’s t-test showing differences in the Shannon diversity index and Chao abundance index between the three enterotype subgroups; **p < 0.01; ***p < 0.001. **D** Spearman’s correlation heatmaps showing relationships between clinical parameters and the relative abundance of dominant OTUs among surviving sepsis patients (*n* = 33). Distinct colors represent correlation level; *p < 0.05; **p < 0.01. **E**, **F** Results of LefSe used to identify essential differences in bacterial abundance (family to species level) between sepsis patients with (*n* = 12) and without (*n* = 26) complications during the ICU stay (**E**), and between surviving (*n* = 33) and non-surviving (*n* = 5) patients (**F**). Only taxa with a significant LDA threshold value of > 3 are shown.

**Additional file 6: Figure S4.** Alterations of the gut microbiome and intestinal transcriptome in septic rats. **A** Student’s t-test showing no significant difference in the Shannon diversity index between septic and healthy rats (*n* = 6 per group). **B** KEGG pathway enrichment analysis of DEGs upregulated (left) and downregulated (right) in the septic ileum (*n* = 6) relative to their expression in the ileum of HCs. The top 15 pathways are shown. **C** Spearman’s correlation was conducted to evaluate associations between the top 50 central DEGs (identified by BC value) and differentially expressed OTUs (LDA > 2) in the ileum of septic rats (*n* = 6). The color gradient corresponds to the R value, where red represents the highest positive correlation and blue represents the lowest.

**Additional file 7: Figure S5**. Sepsis-induced alteration of tryptophan metabolism. Sepsis-related metabolites are shown in color.

**Additional file 8: Table S1.** Clinical characteristics of the derivation and validation group of sepsis patients featured in the metabolic analysis.

**Additional file 9: Table S2.** Covariates that influenced the gut microbiota of sepsis patients.

**Additional file 10: Table S3.** Clinical characteristics of sepsis patients categorized by enterotype.

**Additional file 11: Table S4.** Linear regression between OTU773 and the ICU stay of surviving sepsis patients.

## Data Availability

The datasets used and/or analyzed in the current study are available from the corresponding author upon reasonable request.

## Abbreviations

AhR: Aryl hydrocarbon receptor
APACHE II: Acute Physiology and Chronic Health Evaluation II
BC: Betweenness centrality
CLP: Cecal ligation and puncture
DEG: Differentially expressed gene
ECM: Extracellular matrix
HC: Healthy control
ICU: Intensive care unit
LEfSe: Linear discriminant analysis effect size
LDA: Linear discriminant analysis
OTU: Operational taxonomic unit
PPAR: Peroxisome proliferator-activated receptor
PCA: Principal component analysis
PCoA: Principal coordinates analysis
PXR: Pregnane X receptor
SCFA: Short chain fatty acid
SOFA: Sequential Organ Failure Assessment
STRING: Search Tool for the Retrieval of Interacting Genes/Proteins
VIP: Variable importance in projection
