# Supplementary material for: Altered intestinal microbiome and metabolome correspond to the clinical outcome of sepsis
Source: Crit Care. 2023 Mar 28;27:127. doi: 10.1186/s13054-023-04412-x (PMC10044080; doi:10.1186/s13054-023-04412-x)
Supplement: Supplementary file 1 — Additional file 1: Supplemental methods [file 13054_2023_4412_MOESM1_ESM.docx]

**Supplemental methods**

**Fecal DNA extraction and PCR amplification**

Total microbial genomic DNA was extracted from stool samples using the E.Z.N.A.® soil DNA Kit (Omega Bio-tek, Norcross, GA, U.S.) according to manufacturer’s instructions. The quality and concentration of DNA were determined by 1.0% agarose gel electrophoresis and a Nano Drop® ND-2000 spectrophotometer (Thermo Scientific Inc., USA) and kept at -80 ℃ prior to further use. The hypervariable region V3-V4 of the bacterial 16S rRNA gene were amplified with primer pairs 338F (5'-ACTCCTACGGGAGGCAGCAG-3') and 806R (5'-GGACTACHVGGGTWTCTAAT-3') by an ABI Gene Amp® 9700 PCR thermocycler (ABI, CA, USA). The PCR reaction mixture including 4 μL 5 × Fast Pfu buffer, 2 μL 2.5 mM dNTPs, 0.8 μL each primer (5 μM), 0.4 μL Fast Pfu polymerase, 10 ng of template DNA, and ddH2O to a final volume of 20 µL. PCR amplification cycling conditions were as follows: initial denaturation at 95 ℃ for 3 min, followed by 27 cycles of denaturing at 95 ℃ for 30 s, annealing at 55 ℃ for 30 s and extension at 72 ℃ for 45 s, and single extension at 72 ℃ for 10 min, and end at 4 ℃. All samples were amplified in triplicate. The PCR product was extracted from 2% agarose gel and purified using the AxyPrep DNA Gel Extraction Kit (Axygen Biosciences, Union City, CA, USA) according to manufacturer’s instructions and quantified using Quantus™ Fluorometer (Promega, USA).

**Illumina MiSeq sequencing and Data processing**

Purified amplicons were pooled in equimolar amounts and paired-end sequenced on an Illumina MiSeq PE300 platform/NovaSeq PE250 platform (Illumina, San Diego, USA) according to the standard protocols by Majorbio Bio-Pharm Technology Co. Ltd. (Shanghai, China).

Raw FASTQ files were de-multiplexed using an in-house perl script, and then quality-filtered by fastp version 0.19.6 ^[1]^ and merged by FLASH version 1.2.7 ^[2]^ with the following criteria: (i) the 300 bp reads were truncated at any site receiving an average quality score of <20 over a 50 bp sliding window, and the truncated reads shorter than 50 bp were discarded, reads containing ambiguous characters were also discarded; (ii) only overlapping sequences longer than 10 bp were assembled according to their overlapped sequence. The maximum mismatch ratio of overlap region is 0.2. Reads that could not be assembled were discarded; (iii) Samples were distinguished according to the barcode and primers, and the sequence direction was adjusted, exact barcode matching, 2 nucleotide mismatch in primer matching. Then the optimized sequences were clustered into operational taxonomic units (OTUs) using UPARSE 7.1 ^[3,4]^ with 97% sequence similarity level. The most abundant sequence for each OTU was selected as a representative sequence. The OTU table was manually filtered, i.e., chloroplast sequences in all samples were removed. To minimize the effects of sequencing depth on alpha and beta diversity measure, the number of 16S rRNA gene sequences from each sample were rarefied to 20,000, which still yielded an average Good’s coverage of 99.09%, respectively.

The taxonomy of each OTU representative sequence was analyzed by RDP Classifier version 2.2 ^[5]^ against the 16S rRNA gene database using confidence threshold of 0.7. The metagenomic function was predicted by PICRUSt2 (Phylogenetic Investigation of Communities by Reconstruction of Unobserved States) ^[6]^ based on OTU representative sequences. PICRUSt2 is a software containing a series of tools as follows: HMMER was used to aligns OTU representative sequences with reference sequences. EPA-NG and Gappa were used to put OTU representative sequences into a reference tree. The castor was used to normalize the 16S gene copies. MinPath was used to predict gene family profiles, and locate into the gene pathways. Entire analysis process was accord to protocols of PICRUSt2.

**Metabolite profiling from stool samples**

**Metabolite Extraction**

50 mg solid sample were accurately weighed, and the metabolites were extracted using a 400 µL methanol: water (4:1, v/v) solution with 0.02 mg/mL L-2-chlorophenylalanin as internal standard. The mixture was allowed to settle at -10 ℃ and treated by High throughput tissue crusher Wonbio-96c (Shanghai wanbo biotechnology co., LTD) at 50 Hz for 6 min, then followed by ultrasound at 40 kHz for 30 min at 5 ℃. The samples were placed at -20 ℃ for 30 min to precipitate proteins. After centrifugation at 13000 g at 4 ℃ for 15 min, the supernatant was carefully transferred to sample vials for LC-MS/MS analysis.

**Quality control sample**

As a part of the system conditioning and quality control process, a pooled quality control sample (QC) was prepared by mixing equal volumes of all samples. The QC samples were disposed and tested in the same manner as the analytic samples. It helped to represent the whole sample set, which would be injected at regular intervals (every 10 samples) in order to monitor the stability of the analysis.

**UHPLC-MS/MS analysis.**

The instrument platform for this LC-MS analysis is UHPLC-Q Exactive HF-X system of Thermo Fisher Scientific.

Chromatographic conditions: 2μL of sample was separated by HSS T3 column (100 mm × 2.1 mm i.d., 1.8 μm) and then entered into mass spectrometry detection. The mobile phases consisted of 0.1% formic acid in water: acetonitrile (95:5, v/v) (solvent A) and 0.1% formic acid in acetonitrile: isopropanol: water (47.5:47.5:5, v/v) (solvent B). The solvent gradient changed according to the following conditions: from 0 to 3.5 min, 0% B to 24.5% B (0.4 mL/min); from 3.5 to 5 min, 24.5% B to 65% B (0.4 mL/min); from 5 to 5.5 min, 65% B to 100% B (0.4 mL/min); from 5.5to 7.4 min, 100% B to 100% B (0.4 mL/min to 0.6 mL/min); from 7.4 to 7.6 min, 100% B to 51.5% B (0.6 mL/min); from 7.6 to 7.8 min, 51.5% B to 0% B (0.6 mL/min to 0.5 mL/min); from 7.8 to 9 min, 0% B to 0% B (0.5 mL/min to 0.4 mL/min);from 9 to 10 min, 0% B to 0% B (0.4 mL/min) for equilibrating the systems. The sample injection volume was 2 µL and the flow rate was set to 0.4 mL/min. The column temperature was maintained at 40 ℃. During the period of analysis, all these samples were stored at 4 ℃.

MS conditions: The mass spectrometric data was collected using a Thermo UHPLC -Q Exactive HF-X Mass Spectrometer equipped with an electrospray ionization (ESI) source operating in either positive or negative ion mode. The optimal conditions were set as followed: heater temperature, 425 ℃; Capillary temperature, 325 ℃; sheath gas flow rate, 50 arb; Aux gas flow rate, 13 arb; ion-spray voltage floating (ISVF), -3500V in negative mode and 3500V in positive mode, respectively; Normalized collision energy, 20-40-60V rolling for MS/MS. Full MS resolution was 60000, and MS/MS resolution was 7500. Data acquisition was performed with the Data Dependent Acquisition (DDA) mode. The detection was carried out over a mass range of 70-1050 m/z.

**Data preprocessing and annotation**

After the mass spectrometry detection is completed, the raw data of LC/MS is preprocessed by Progenesis QI (Waters Corporation，Milford, USA) software, and a three-dimensional data matrix in CSV format is exported. The information in this three-dimensional matrix includes: sample information, metabolite name and mass spectral response intensity. Internal standard peaks, as well as any known false positive peaks (including noise, column bleed, and derivatized reagent peaks), were removed from the data matrix, deredundant and peak pooled. At the same time, the metabolites were searched and identified, and the main database was the HMDB (http://www.hmdb.ca/) Metlin (https://metlin.scripps.edu/) and Majorbio Database.

The data after the database search is uploaded to the Majorbio cloud platform (https://cloud.majorbio.com) for data analysis. Metabolic features detected at least 80 % in any set of samples were retained. After filtering, minimum metabolite values were imputed for specific samples in which the metabolite levels fell below the lower limit of quantitation and each Metabolic features were normalized by sum. In order to reduce the errors caused by sample preparation and instrument instability, the response intensity of the sample mass spectrum peaks was normalized by the sum normalization method, and the normalized data matrix was obtained. At the same time, variables with relative standard deviation (RSD) >30% of QC samples were removed, and log10 alogorithmization was performed to obtain the final data matrix for subsequent analysis. The raw data of metabolome could be found at URL: www.ebi.ac.uk/metabolights/MTBLS6429.

**RNA isolation and sequencing**

Total RNA was isolated from the small and large intestine of rats using the MiniBEST Universal RNA Extraction Kit (TaKaRa, Kusatsu, Shiga, Japan) and RNA-sequencing was done by Shanghai Personalbio (Shanghai, China). Transcriptomic analysis was performed by Majorbio (Shanghai, China). Quality control of raw sequencing reads was performed with MiSeq internal software during base calling. High-quality clean reads were obtained after quality, ambiguity, and length trimming. The sequencing reads were mapped to the GRCm38.p5 genome with HISAT2 software ^[7]^. Transcript assemblies were generated using StringTie ^[8]^, transcripts were annotated with Cluster of Orthologous Groups (COG) using DIAMOND ^[9]^, and protein functions were assigned according to their locus tags ^[10]^. Differential gene expression analysis was performed using both the edgeR ^[11]^ and DESeq2 ^[12]^ R packages, and Kal’s test with false discovery rate (FDR) correction was applied. An adjusted P value < 0.05 was accepted as indicating differentially expressed genes after Benjamini-Hochberg post hoc correction. The functional groups comprising differentially expressed genes were analyzed based on Gene Ontology (GO) annotation and Kyoto Encyclopedia of Genes and Genomes (KEGG) ^[13]^. A principal component analysis (PCA) plot was generated and used to assess reproducibility.

**Reference**

[1] fastp: an ultra-fast all-in-one FASTQ preprocessor.

[2] FLASH: fast length adjustment of short reads to improve genome assemblies.

[3] UPARSE: highly accurate OTU sequences from microbial amplicon reads.

[4] Taxonomic Note: A Place for DNA-DNA Reassociation and 16S rRNA Sequence Analysis in the Present Species Definition in Bacteriology.

[5] Naive Bayesian classifier for rapid assignment of rRNA sequences into the new bacterial taxonomy.

[6] PICRUSt2 for prediction of metagenome functions.

[7] HISAT: a fast spliced aligner with low memory requirements.

[8] StringTie enables improved reconstruction of a transcriptome from RNA-seq reads.

[9] Fast and sensitive protein alignment using DIAMOND.

[10] The COG database: a tool for genome-scale analysis of protein functions and evolution.

[11] edgeR: a Bioconductor package for differential expression analysis of digital gene expression data.

[12] Moderated estimation of fold change and dispersion for RNA-seq data with DESeq2.

[13] KEGG: new perspectives on genomes, pathways, diseases and drugs.
