# Supplementary figures and images for: Altered intestinal microbiome and metabolome correspond to the clinical outcome of sepsis
Source: Crit Care. 2023 Mar 28;27:127. doi: 10.1186/s13054-023-04412-x (PMC10044080; doi:10.1186/s13054-023-04412-x)

(A)

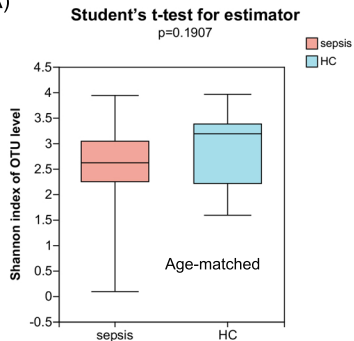

(B)

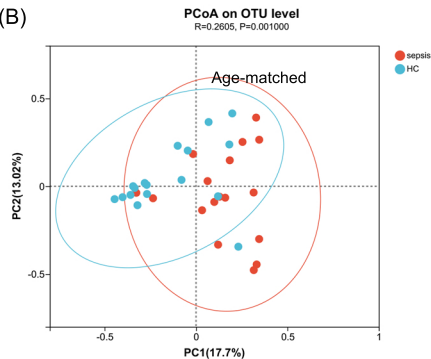

(C)

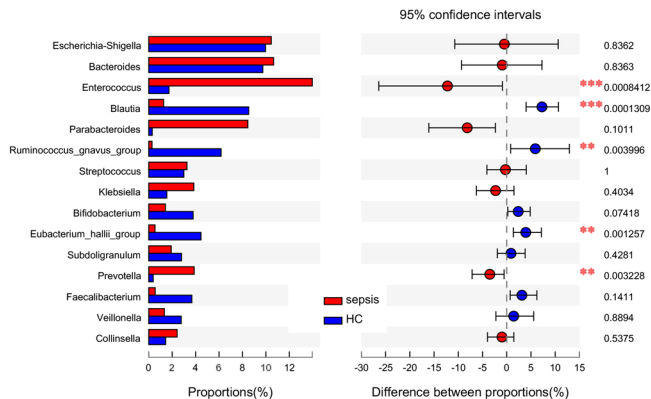

(D)

### Community barplot analysis

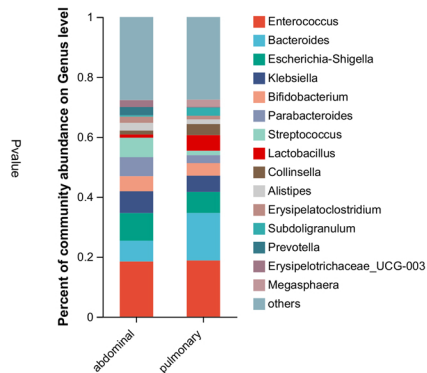

Supplement: Supplementary file 3 — Additional file 3: Figure S1. Comparison of microbial alterations in the sepsis patients and the HCs after age matching (n = 17 per group). A PCoA for the sepsis and HC group samples, with plots based on the Bray–Curtis distance. Each point represents one sample and the colors represent different groups. The results of the ANOSIM test to compare dissimilarity indexes among samples are shown above the plots. B Wilcoxon rank sum tests performed to analyze between-group differences in the main bacterial load at the genus level. C Average relative proportions of genera in patients according to origin of infection. [file 13054_2023_4412_MOESM3_ESM.pdf]

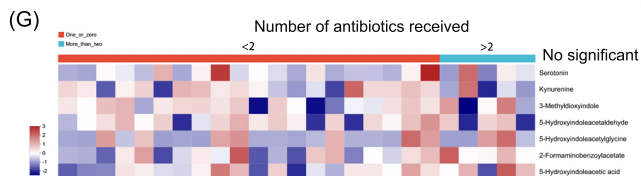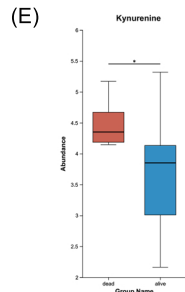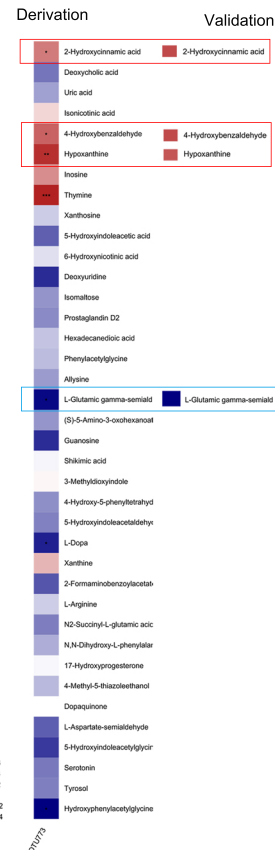

Supplement: Supplementary file 4 — Additional file 4: Figure S2. Metabolic profiles of sepsis patients and their association with gut microbiota and disease severity. A PCA of the stool samples from sepsis patients and HCs, plotted in negative-ion mode. B Principal component analysis (PCA) for septic samples receiving different numbers of antibiotics in positive-ion mode. C Spearman’s correlation heatmap showing the test results between differential species at the OTU level and altered sepsis-associated metabolites among samples in the validation group (n = 12). Distinct colors represent correlation level; *p < 0.05; **p < 0.01; ***p < 0.001. D Heatmap showing the significant differential metabolites between surviving (n = 21) and non-surviving (n = 4) patients in the derivation group. E Wilcoxon rank sum test performed to compare the abundance of fecal kynurenine between the surviving and nonsurviving patients. F Spearman’s correlation between the OTU773 (B. vulgatus) load and the abundance of sepsis-associated metabolites among samples in the derivation group (n = 25). Confirmation of the correlations between OTU773 and four metabolites (2-hydroxycinnamic acid, 4-hydroxybenzaldehyde, hypoxanthine, and L-glutamic gamma-semiarid) in the validation group (n = 12). Distinct colors represent correlation level; *p < 0.05; **p < 0.01; ***p < 0.001. [file 13054_2023_4412_MOESM4_ESM.pdf]

(A)

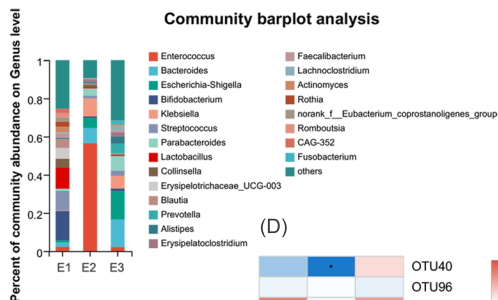

(B)

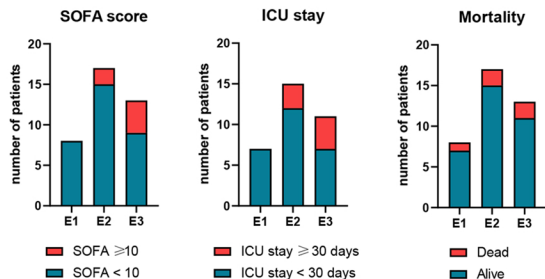

(C)

### Enterotype-Shannon diversity

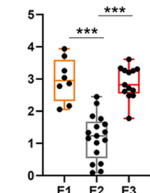

### Enterotype-Chao abundance

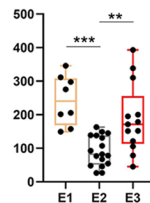

(D)

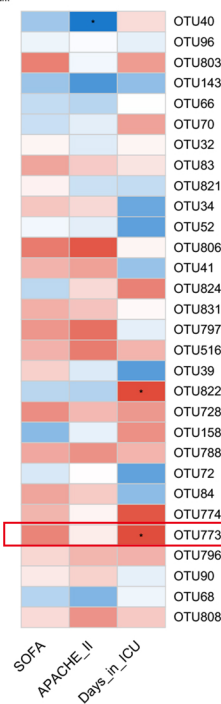

(E)

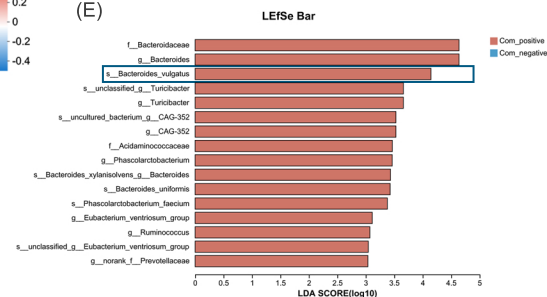

(F)

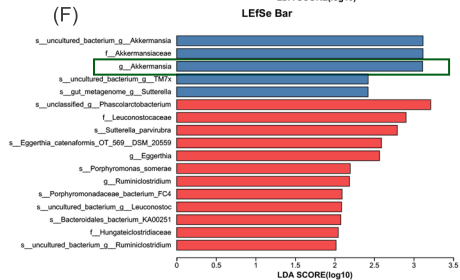

Supplement: Supplementary file 5 — Additional file 5: Figure S3. Possible relationships between enterotype and clinical severity of sepsis. A Average relative proportions of genera in each enterotype. B Column diagrams showing the proportions of sepsis patients with a SOFA score ≥ 10, an ICU stay ≥ 30 days, and death. C Student’s t-test showing differences in the Shannon diversity index and Chao abundance index between the three enterotype subgroups; **p < 0.01; ***p < 0.001. D Spearman’s correlation heatmaps showing relationships between clinical parameters and the relative abundance of dominant OTUs among surviving sepsis patients (n = 33). Distinct colors represent correlation level; *p < 0.05; **p < 0.01. E, F Results of LefSe used to identify essential differences in bacterial abundance (family to species level) between sepsis patients with (n = 12) and without (n = 26) complications during the ICU stay (E), and between surviving (n = 33) and non-surviving (n = 5) patients (F). Only taxa with a significant LDA threshold value of > 3 are shown. [file 13054_2023_4412_MOESM5_ESM.pdf]

(C)

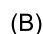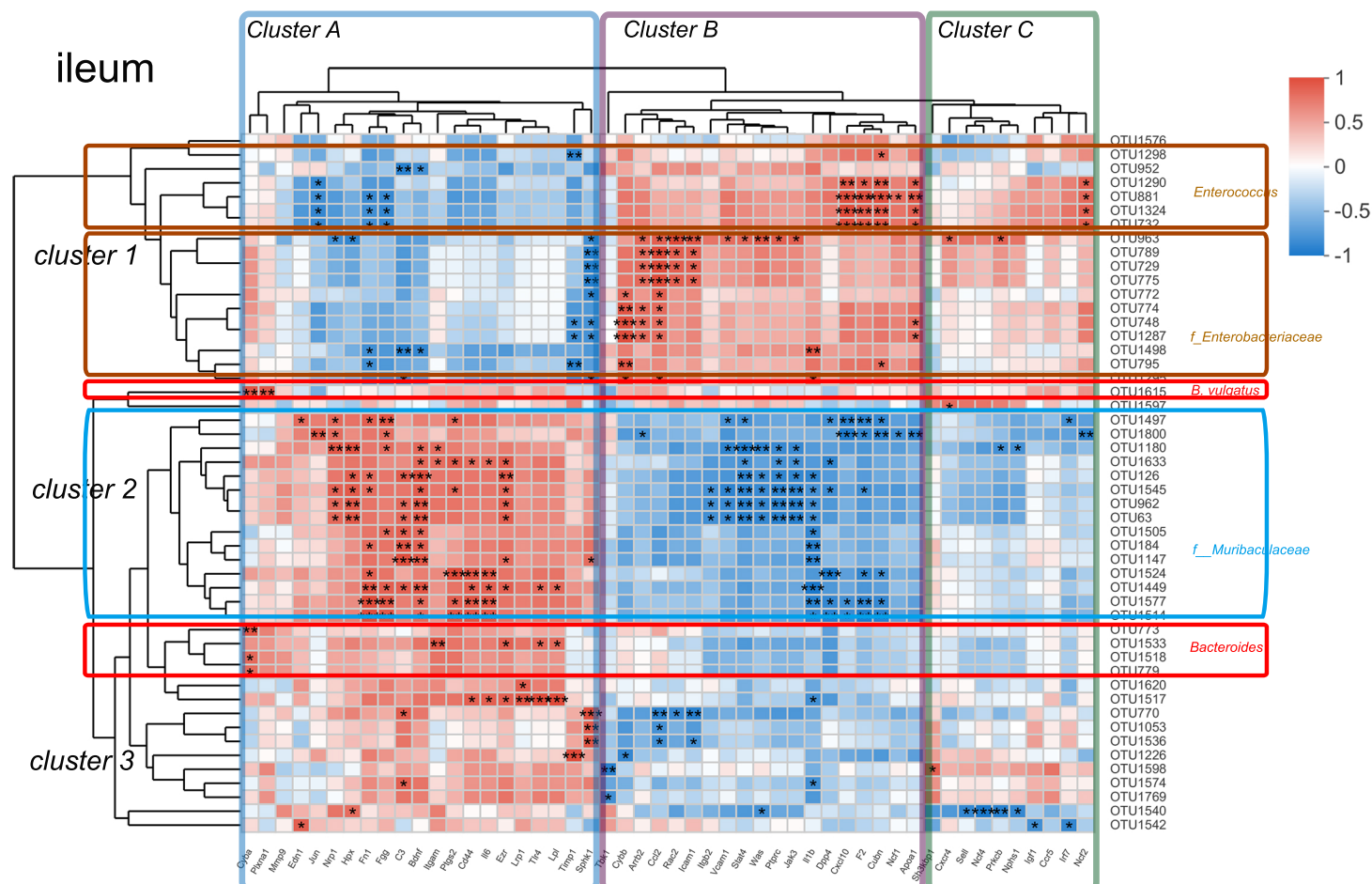

Supplement: Supplementary file 6 — Additional file 6: Figure S4. Alterations of the gut microbiome and intestinal transcriptome in septic rats. A Student’s t-test showing no significant difference in the Shannon diversity index between septic and healthy rats (n = 6 per group). B KEGG pathway enrichment analysis of DEGs upregulated (left) and downregulated (right) in the septic ileum (n = 6) relative to their expression in the ileum of HCs. The top 15 pathways are shown. C Spearman’s correlation was conducted to evaluate associations between the top 50 central DEGs (identified by BC value) and differentially expressed OTUs (LDA > 2) in the ileum of septic rats (n = 6). The color gradient corresponds to the R value, where red represents the highest positive correlation and blue represents the lowest. [file 13054_2023_4412_MOESM6_ESM.pdf]

## TRYPTOPHAN METABOLISM

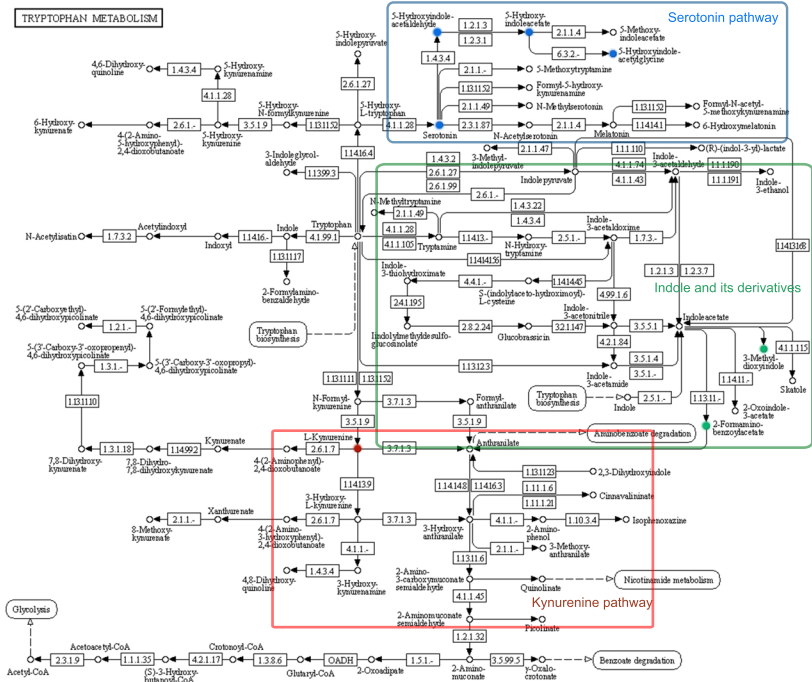

Supplement: Supplementary file 7 — Additional file 7: Figure S5. Sepsis-induced alteration of tryptophan metabolism. Sepsis-related metabolites are shown in color. [file 13054_2023_4412_MOESM7_ESM.pdf]
