# Supplementary material for: Altered intestinal microbiome and metabolome correspond to the clinical outcome of sepsis
Source: Crit Care. 2023 Mar 28;27:127. doi: 10.1186/s13054-023-04412-x (PMC10044080; doi:10.1186/s13054-023-04412-x)
Supplement: Supplementary file 8 — Additional file 8: Table S1. Clinical characteristics of the derivation and validation group of sepsis patients featured in the metabolic analysis. [file 13054_2023_4412_MOESM8_ESM.docx]

**Table S1 Clinical characteristics of sepsis patients of two groups in metabolic analysis.**

|  | Derivation group  （n=25） | Validation group  （n=12） | P |
| --- | --- | --- | --- |
| Age, mean ± SD, years | 63.24±16.65 | 58±14.83 | 0.360 |
| Gender, male/female | 11/14 | 10/2 | 0.035* |
| BMI, mean ± SD | 22.23±4.55 | 22.67±3.46 | 0.771 |
| Origin of infection |  |  |  |
| Abdominal | 10(40%) | 8(66.7%) | 0.170 |
| Pulmonary | 8(32%) | 2(16.7%) | 0.445 |
| Complication occurred over the ICU period | 8(32%) | 3(25%) | 1.000 |
| SOFA, mean ± SD | 6.56±3.27 | 5.08±3.06 | 0.198 |
| APAHCE-II, mean ± SD | 13.04±6.29 | 8.58±7.62 | 0.068 |
| Samples collected within 48 hours of treatment | 13(52%) | 7(58.3%) | 0.498 |
| Mortality within 90 days, frequency (percentage) | 4 (16%) | 1 (8.3%) | 0.470 |
| ICU stay, days | 18.04±14.26 | 12.17±8.42 | 0.127 |

Abbreviations: APACHE; acute physiology and chronic health evaluation; SOFA; sequential organ failure assessment.

Numerical data are compared by student’s t-test, and categorical data are compared by Fisher’s exact test.
