# Supplementary material for: Altered intestinal microbiome and metabolome correspond to the clinical outcome of sepsis
Source: Crit Care. 2023 Mar 28;27:127. doi: 10.1186/s13054-023-04412-x (PMC10044080; doi:10.1186/s13054-023-04412-x)
Supplement: Supplementary file 9 — Additional file 9: Table S2. Covariates that influenced the gut microbiota of sepsis patients. [file 13054_2023_4412_MOESM9_ESM.docx]

**Table S2 Covariates influencing the gut microbiome and metabolome of sepsis patients.**

|  | | Microbiome | | | | | | | | | Metabolome ^f^ | | |
| --- | --- | --- | --- | --- | --- | --- | --- | --- | --- | --- | --- | --- | --- |
|  | |  | Shannon diversity | | OTU808(%)  (*E. faecium*) | | OTU773(%)  (*B. vulgatus*) | | PC1^a^ | PC2 |  | PC1  Positive ion | PC1  Negative ion |
|  | |  | Mean±SD^b^ | P^c^ | Mean±SD | P | Mean±SD | P | P | |  | P | |
| **Age** | |  |  | | | | | | | |  |  | |
| Young (<60) | | N=16 | 2.36±0.94 | 0.309 | 19.5±31.1 | 0.280 | 2.46±3.86 | 0.780 | 0.178 | 0.989 | N=9 | 0.245 | 0.204 |
| Old (≥60) | | N=22 | 1.99±1.14 |  | 31.6±35.2 |  | 2.06±4.64 |  |  |  | N=16 |  |  |
| **Gender** | |  |  | | | | | | | |  |  | |
| Male | | N=22 | 2.31±1.05 | 0.272 | 22.6±29.4 | 0.406 | 1.94±3.47 | 0.631 | 0.620 | 0.299 | N=11 | 0.604 | 0.686 |
| Female | | N=16 | 1.92±1.08 |  | 31.9±39.2 |  | 2.63±5.31 |  |  |  | N=14 |  |  |
| **BMI** | |  |  | | | | | | | |  |  |  |
| BMI<25 | | N=29 | 1.88±1.00 | **0.005*** | 33.2±35.7 | **0.026*** | 1.48±3.19 | 0.051 | **0.017*** | 0.318 | N=20 | 0.876 | 0.648 |
| BMI≥25 | | N=9 | 3.00±0.85 |  | 4.94±9.71 |  | 4.65±6.37 |  |  |  | N=5 |  |  |
| **Time interval from medical treatment to specimen collection** | | | | | | | | | | |  |  | |
| <48h (n=21) | | N=21 | 2.14±1.13 | 0.972 | 31.2±35.4 | 0.350 | 2.20±3.85 | 0.969 | 0.481 | 0.685 | N=13 | 0.768 | 0.656 |
| >48h (n=17) | | N=17 | 2.15±1.01 |  | 20.8±31.5 |  | 2.26±4.89 |  |  |  | N=12 |  |  |
| **Origin of infection** | |  |  | | | | | | | |  |  | |
| Abdominal (n=19) | | N=19 | 2.30±0.74 | 0.546 | 18.5±25.1 | 0.975 | 2.34±4.64 | 0.808 | 0.909 | 0.604 | N=10 | 0.302 | 0.235 |
| Pulmonary (n=10) | | N=10 | 2.51±1.09 |  | 18.8±29.2 |  | 2.78±4.44 |  |  |  | N=8 |  |  |
| **Antibiotics type^d^** | |  |  | | | | | | | |  |  | |
| Carbapenem | Y^e^ | N=20 | 2.24±1.12 | 0.678 | 26.5±34.2 | 0.963 | 2.82±5.17 | 0.427 | 0.952 | 0.147 | N=12 | 0.427 | 0.318 |
|  | N | N=17 | 2.10±1.02 |  | 35.1±26.0 |  | 1.67±3.10 |  |  |  | N=13 |  |  |
| Cephalosporin | Y | N=10 | 1.90±1.10 | 0.339 | 30.2±29.4 | 0.680 | 1.34±2.56 | 0.424 | 0.636 | 0.436 | N=7 | 0.997 | 0.555 |
|  | N | N=27 | 2.28±1.05 |  | 24.8±32.6 |  | 2.64±3.81 |  |  |  | N=18 |  |  |
| Quinolones | Y | N=5 | 2.63±1.30 | 0.314 | 27.3±33.4 | 0.656 | 2.25±4.38 | 0.909 | 0.382 | 0.374 | N=3 | 0.142 | 0.068 |
|  | N | N=32 | 2.11±1.03 |  | 19.8±41.8 |  | 2.50±4.46 |  |  |  | N=22 |  |  |
| Metronidazole | Y | N=5 | 2.26±0.74 | 0.798 | 13.1±12.8 | 0.086 | 1.89±1.88 | 0.854 | 0.453 | 0.584 | N=0 | - | - |
|  | N | N=32 | 2.13±1.11 |  | 28.5±35.4 |  | 2.28±4.56 |  |  |  | N=25 |  |  |
| **Number of antibiotics** | | |  | | | | | | | |  |  | |
| 0 | | N=3 | 1.91±0.75 | 0.596^g^ | 26.5±43.6 | 0.410 | 0.11±0.00 | 0.426 | 0.598 | 0.204 | N=2 | **0.016*** | 0.052 |
| 1 | | N=24 | 2.13±1.14 |  | 29.6±36.4 |  | 2.91±5.01 |  |  |  | N=14 |  |  |
| ≥2 | | N=10 | 2.35±1.02 |  | 19.2±28.4 |  | 1.58±3.06 |  |  |  | N=9 |  |  |
| **Proton-pump inhibitors** | | |  | | | | | | | |  |  | |
| Y | | N=15 | 1.93±1.19 | 0.303 | 32.3±38.2 | 0.399 | 1.88±3.92 | 0.696 | 0.523 | 0.765 | N=14 | 0.247 | 0.790 |
| N | | N=23 | 2.29±0.98 |  | 22.7±30.7 |  | 2.45±4.58 |  |  |  | N=11 |  |  |

1. PC1 and PC2 represent the two most informative principal coordinates (PCs) of the principal coordinate analysis (PCoA), demonstrating the gut microbiota or metabolic structure of sepsis and HC samples. b. SD, standard deviation; c. The difference between two groups were identified by Student’s t-test. d. The record of antibiotic treatment for S31 was missed; e. Y=yes, N=no. f. The PC1 and PC2 were obtained in metabolome of the derivation group. g. The P value was obtained from comparison between “1” and “≥2” group. *P < 0.05
