# Supplementary material for: Altered intestinal microbiome and metabolome correspond to the clinical outcome of sepsis
Source: Crit Care. 2023 Mar 28;27:127. doi: 10.1186/s13054-023-04412-x (PMC10044080; doi:10.1186/s13054-023-04412-x)
Supplement: Supplementary file 10 — Additional file 10: Table S3. Clinical characteristics of sepsis patients categorized by enterotype. [file 13054_2023_4412_MOESM10_ESM.docx]

**Table S3** Clinical characteristics of patients with sepsis of different enterotypes.

| Enterotype | SOFA score | | APACHE II score | | ICU stay time | | Mortality | |
| --- | --- | --- | --- | --- | --- | --- | --- | --- |
|  | <10 | ≥10 | <18 | ≥18 | <30d | ≥30d | Alive | Dead |
| E1+E2 (n=25) | 23(92%) | 2(8%) | 22(88%) | 3(12%) | 19(86%) | 3(14%) | 22(88%) | 3(12%) |
| E3 (n=13) | 9(69%) | 4(31%) | 7(54%) | 6(46%) | 7(64%) | 4(36%) | 11(85%) | 2(15%) |
| Fisher's exact test, P | 0.195 | | **0.040*** | | 0.186 | | 1.000 | |

P values were calculated using Fisher's exact test.

SOFA, Sequential Organ Failure Assessment; APACHE II, Acute Physiology and Chronic Health Evaluation II; E1-3, enterotype 1-3
