# Supplementary material for: Altered intestinal microbiome and metabolome correspond to the clinical outcome of sepsis
Source: Crit Care. 2023 Mar 28;27:127. doi: 10.1186/s13054-023-04412-x (PMC10044080; doi:10.1186/s13054-023-04412-x)
Supplement: Supplementary file 11 — Additional file 11: Table S4. Linear regression between OTU773 and the ICU stay of surviving sepsis patients. [file 13054_2023_4412_MOESM11_ESM.docx]

**Table S4**. Linear regression between OTU773 and the ICU stay of those surviving sepsis patients.

| Model | Included variables | Multivariable Analysis | |
| --- | --- | --- | --- |
|  |  | Standard regression coefficient | P value |
| Model 1 | OTU773 | 0.465 | **0.011*** |
|  | OTU808 | -0.005 | 0.975 |
| Model 2 | OTU773 | 0.453 | **0.037*** |
|  | OTU808 | -0.005 | 0.983 |
|  | Age >60 | -0.007 | 0.976 |
|  | BMI >25 | -0.111 | 0.628 |
|  | Gender | -0.097 | 0.635 |
|  | Origin of infection | 0.156 | 0.475 |
|  | Use of carbapenems | 0.005 | 0.979 |
|  | Use of cephalosporins | 0.088 | 0.737 |
|  | Use of quinolones | -0.333 | 0.134 |
|  | Use of metronidazole | 0.146 | 0.462 |
|  | Use of proton-pump inhibitors | -0.207 | 0.330 |
|  | Time interval from treatment to specimen collection | -0.058 | 0.788 |

Model 2 includes such variables that potentially affect the structure of the microbiome. *P< 0.05,
